# Impact of Dealcoholization by Osmotic Distillation on Metabolic Profile, Phenolic Content, and Antioxidant Capacity of Low Alcoholic Craft Beers with Different Malt Compositions

**DOI:** 10.1021/acs.jafc.1c00679

**Published:** 2021-04-15

**Authors:** Rita Petrucci, Paola Di Matteo, Anatoly P. Sobolev, Loredana Liguori, Donatella Albanese, Noemi Proietti, Martina Bortolami, Paola Russo

**Affiliations:** † Department of Basic and Applied Sciences for Engineering, Sapienza University of Rome, Via del Castro Laurenziano 7, 00161 Rome, Italy; ‡ Department of Chemical Engineering Materials Environment, Sapienza University of Rome, Via Eudossiana 18, 00184 Rome, Italy; § “Segre-Capitani” Magnetic Resonance Laboratory, Institute for Biological Systems, National Research Council (CNR), via Salaria km 29.300, Monterotondo, 00015 Rome, Italy; ∥ Department of Industrial Engineering, University of Salerno, Via Giovanni Paolo II 132, 84084 Fisciano, Salerno, Italy

**Keywords:** beer antioxidants, hydroxybenzoic acids, hydroxycinnamic acids, flavonols, oligosaccharides, amino acids, organic acids, HPLC-ESI-MS/MS, NMR

## Abstract

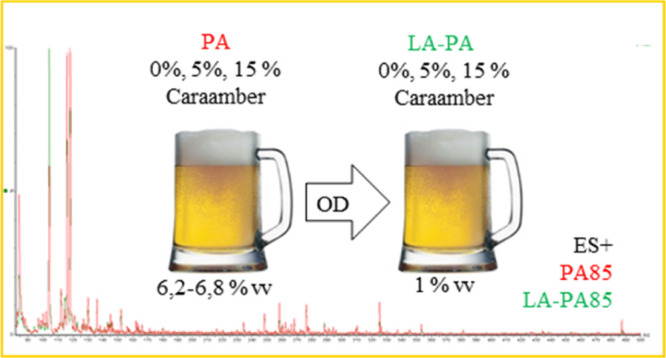

Beer antioxidants originate mainly from malts, classified as colored, caramel, and roasted, according to the malting process. This study aimed to characterize, in terms of phenolic antioxidants, three types of Pale Ale craft beers brewed using increasing percentage of dark malt (0, 5, and 15% Caraamber malt, called PA100, PA95, PA85, respectively) and to evaluate the impact of dealcoholization by osmotic distillation (OD) on the same antioxidants. All the alcoholic (PA, 6.2–6.8 vol %) and low alcoholic (LA-PA, 1 vol %) beers were analyzed by HPLC-ESI-MS/MS, total phenolic content (TPC), and antioxidant activity (AA): similar phenolic profiles were evidenced and 43 compounds identified or tentatively identified. Some differences were found among PA100, PA95, and PA85: PA85 was richer in free phenolic compounds (10.55 mg/L) and had a higher TPC (463.7 GAE mg/L) and AA (852.1 TE mg/L). LA-PA beers showed the same phenolic profile and similar TPC and AA compared to PA beers; however, there were some differences regarding LA-PA85 (5.91 mg/L). Dealcoholization by OD seemed to weakly affect the phenolic fraction. ESI-MS/MS infusion experiments evidenced oligosaccharides, small organic acids, and amino acids, whose presence was confirmed and quantitated by NMR: besides ethanol and other alcohols, weak to strong loss of low-molecular-weight metabolites was evidenced in LA-PA beers.

## Introduction

Beer is the biggest segment in the market of alcoholic drinks worldwide. Nowadays, the beer consumption is increasing globally and, according to recent reports, the beer market will be over 730 billion USD by 2022.^1^ Inside this market, craft beer demand plays an interesting role, with forecast sales of 500 billion USD by 2025.^2^ The popularity of beer is mainly due to its sensory profile (i.e., appearance, taste, aroma, and foam) combined with health benefits (i.e., antioxidant properties and low calorie content),^3−6^ and the latest challenge of brewers is to preserve taste and benefit in beer with a drastically reduced alcohol content.^7−9^ Taking into account also the legislative and religious restrictions on alcohol consumption, the market of low alcoholic beer and alcohol-free beer is expected to grow at a high rate over the next 5 years.^9^ The alcohol content in low alcoholic or alcohol-free beer depends on regulations and varies with countries: in Europe, according to Regulation (EU) No 1169/2011 (25.10.2011), an alcohol content by volume (ABV) below 1.2% is requested to be labeled as low alcoholic beer, whereas alcohol-free beer must have an ABV of 0.05% or lower.

The production of low alcoholic beer with organoleptic properties similar to those of regular beer^10^ is one of the most difficult tasks. Several technologies have been developed in the last decades, aiming to reduce the alcohol content in beverages.^7,9,11^ Membrane processes seem to be the most promising ones, with the advantages of preserving volatile compounds from thermal damage and of low energy consumption. Among the others, nanofiltration, reverse osmosis, and osmotic distillation (OD) are the most investigated processes, in which a concentration or pressure gradient is the driving force for the alcohol removal. In particular, several studies have been reported on OD.^12−15^ An OD process was recently optimized in order to reduce the aroma compounds loss, and the impact on chemical–physical characteristics (i.e., organic acids, total phenols, foam, and turbidity), volatile profile, and sensory properties was investigated.^12,16,17^

Other studies were focused on beer antioxidants, which exert an important role in taste, aroma, astringency, body, and fullness.^18,19^ Phenolic compounds are known to have good antioxidant properties;^20,21^ they are always present in beer, both as free and bonded forms,^22^ and their contents depend on starting material (such as malt and hops), recipe, and brewing practice.^23−27^

Several methods for the analysis of phenolic compounds in beverages, including beer, by liquid chromatography-tandem mass spectrometry were reported;^28−32^ all of them requiring a sample pretreatment. Moreover, a method for the rapid analysis of free polyphenols in beer without sample pretreatment was recently reported.^33^

To the best of our knowledge, only few studies have been reported on the characterization of craft beer in terms of chemical–physical properties, phenolic acid content, and antioxidant activity (AA).^27^ Similarly, few studies have been reported on the effect of the dealcoholization process on sensory properties and quality.^12,14,16^

In the present work, three types of Pale Ale craft beers were brewed in a small pilot plant, by using different malt composition with increasing caramel malt percentages (0, 5, and 15% Caraamber malt). The beers were successively dealcoholized by OD. All the regular and low alcoholic beers were analyzed by liquid chromatography-tandem mass spectrometry and NMR and tested for total phenolic content (TPC) and antioxidant capacity, with the aim (a) to provide a comprehensive chemical characterization, (b) to evaluate the influence of malt composition on the phenolic profile, and (c) to test the impact of the OD process on the beneficial antioxidant properties.

## Materials and Methods

### Materials

#### Chemicals and Solvents

Gallic acid (GA), *p*-hydroxybenzoic acid (pHBA), *m*-hydroxybenzoic acid (mHBA), 3,4-dihydroxybenzoic acid (PCA, protocathecuic acid), vanillic acid (VA), syringic acid (SyA), *p*-coumaric acid (CuA), caffeic acid (CA), ferulic acid (FA), sinapic acid (SA), 5-caffeoylquinic acid (CQA), quercetin (Q), kampferol (K), rutin (Ru), 3-(trimethylsilyl)propionic-2,2,3,3-*d*_4_ acid sodium salt (TSP), sodium azide, deuterium oxide, and 2,2-diphenyl-1-picrylhydrazyl (DPPH) radical were of analytical grade, acquired from Sigma-Aldrich (Milano, Italy) and used as received. The Folin-Ciocalteu phenol reagent was purchased from Sinopharm Chemical Reagent (Shanghai, China). HPLC grade acetonitrile and methanol were purchased from Carlo Erba (Milano, Italy); HPLC grade water was prepared with the Milli-Q purification system (Millipore, Vimodrone, Italy).

#### Beer Production

The malts (Pale Ale and Caraamber malts) were acquired from Weyermann (Bamberg, Germany); Challenger and East Kent Golding hops and the top-fermenting yeast Safale S-04 Fermentis were provided from P.A.B. Srl–Mr. Malt (Pasian di Prato, Udine, Italy). Three different types of Pale Ale craft beers were produced using Pale Ale and Caraamber malts in the three different percentages Pale Ale 100% (PA100), Pale Ale 95%-Caraamber 5% (PA95), and Pale Ale 85%-Caraamber 15% (PA85), as previously described.^34^ Briefly, the worts of all the beers were produced in a 50 L pilot scale brewery at the Food Technology Laboratory (University of Salerno, Italy), in a water/grist ratio of 4:1 and mashing steps of 60 min at 67 °C and 5 min at 78 °C.

Challenger and East Kent Golding hop pellets were added first and at the end of boiling, respectively. The wort was transferred in a whirlpool and then in a plate heat exchanger until reaching the temperature of 24 °C. Finally, the wort (40 L) was collected in a fermenter and inoculated by dry yeast (0.28 g/L), previously activated in warm water. The fermentation was carried out at 20 ± 2 °C for 7 days. After racking in a new vessel, the green beer was stored at 4.0 ± 0.5 °C for 15 days in order to allow the maturation and clarification phenomena. Finally, the beer was bottled in 660 mL glass bottles.

### Methods

#### Beers Dealcoholization Process

The dealcoholization process was carried out by OD, as previously reported.^12^ In particular, the laboratory plant was equipped with a membrane module with hollow fibers (1.7 × 5.5 MiniModule, Liqui-Cel, Wuppertal, Germany) in which beer and stripper counter flowed in the tubes (flow rate = 0.7 L/min) and in the shell (flow rate = 1.4 L/min), respectively. The process was set at 10 °C through four dealcoholization cycles, useful to reach an alcohol content lower than 1.2% vol. The alcohol content of the regular craft beers and the corresponding low alcoholic craft beers was analyzed, according to Analytica-European Brewing Convention (EBC) methods (2010).^35^

#### HPLC-ESI-MS/MS Analysis of Free Phenolic Compounds

The craft beers as received were degassed for 10 min in an ultrasonic bath, filtered at 0.45 μm, diluted 1:10 with the mobile phase (A/B, 95:5), and injected in triplicate (25 μL) for analysis. Experiments were carried out by an HPLC 1525μ Waters (Milford, MA, USA), using a Waters XBridge C18 (150 × 2.1 mm i.d.) 5 μm analytical column and a Waters Quattro Micro Tandem MS/MS detector with an ESI source (Micromass, Manchester UK). Mass spectral data were acquired in negative ionization (ES−), by using the selected ion recording (SIR) mode. Data acquisition, data handling, and instrument control were performed by MassLynx Software 4.1 v (Data Handling System for Windows, Micromass, UK).

The analysis of free phenolic compounds was carried out as recently reported in the literature.^33^ Briefly, the chromatographic separation was carried out with the gradient 0–1 min, 5% B; 1–20 min, 16.5% B; 20–30 min, 40% B; 30–35 min, 60% B; 35–36 min, 80% B; 36–37 min, 80% B; 37–38 min, 5% B; 38–58 min, 5% B to equilibrate the column; A was MilliQ water/formic acid 0.02% and B was acetonitrile/formic acid 0.02%, flow rate 0.20 mL/min. The detection was carried out by acquiring spectral data in the SIR mode in negative ionization (ES−), using a separated acquisition channel for each different monoisotopic mass corresponding to the deprotonated anion [M – H]^−^ of the searched compounds, in detail: 169 *m*/*z* (GA), 137 *m*/*z* (pHBA and mHBA), 153 *m*/*z* (PCA), 167 *m*/*z* (VA), 197 *m*/*z* (SyA), 179 *m*/*z* (CA), 163 *m*/*z* (CuA), 193 *m*/*z* (FA), 223 *m*/*z* (SA), 353 *m*/*z* (CQA), 301 *m*/*z* (Q), 285 *m*/*z* (K), and 609 *m*/*z* (Ru). The analytical method^33^ has been improved for the quantitation of phenolic compounds in the beers analyzed in the present work, as follows.

A stock solution, containing the 14 standards (STDs) 1 mg/mL in methanol, was diluted with the mobile phase (A/B, 95:5) to the final concentration of 10, 14, 20, 50, and 100 μg/L, and each sample was injected in triplicate (25 μL). The calibration curves of GA, PCA, CA, SyA, and SA were calculated, as previously described for CGA, pHBA, mHBA, VA, CuA, FA, Ru, Q, and K.^33^ Limits of detection (LOD) and quantitation (LOQ) were evaluated by a calibration approach and linear regression and calculated, as reported in the literature.^33,36,37^ Interday precision was evaluated by triplicate injections, in three different days, at three different concentrations (14, 50, and 100 μg/L); intraday precision was evaluated by five injections of a 50 μg/L STD solution. Results were given as percent relative standard deviation (rsd %). Accuracy was evaluated by triplicate injections of a 25 μg/L STD solution, and it was given as percent difference between the nominal concentration and the measured one. Percent recovery was obtained by spiking in duplicate 10, 14, 20, 50, and 100 μg/L STD solution into the 1:10 diluted beer sample and calculated as the ratio of the STD peak area in the spiked solution and in the STD solution. Matrix effect (ME) was evaluated by comparing the matrix-matching calibration curve (10, 14, 20, 50, and 100 μg/L) with the corresponding STD calibration curve. Linearity and sensitivity data are shown in Table S1; precision, accuracy, recovery, and ME data are shown in Table S2.

The free phenolic compounds were identified in beer by matching spectral data and chromatographic retention time (*t*_R_) with STD, resumed in Table 1, and they were quantitated using the corresponding calibration curve. Results (mg/L beer) are reported in Table 2 as mean values ± standard deviation. The total amount (TA, mg/L beer) of free polyphenols for each regular and low alcoholic beer is also reported in Table 2 for a direct comparison.

**Table 1:** Phenolic Compounds in the Regular and Low Alcoholic Craft Beers Analyzed by HPLC-ESI-MS/MS

peak	name	*t*_R_ (min)	[M – H^–^]^−^ (*m*/*z*)	[M-174-H]^−^ (*m*/*z*)	[M-162-H]^−^ (*m*/*z*)
1	unknown	2.87	301		
2	3,4,5-trihydroxybenzoic acid (GA)^a^	3.04	169		
3	caffeic acid-*O*-hexoside I^b^	3.37			179
4	hydroxy-dimethoxybenzoic acid I^b^	3.78	197		
5	hydroxy-methoxybenzoic acid I^b^	4.02	167		
6	unknown	4.12	285		
7	sinapic acid-*O*-hexoside I^b^	4.18			223
8	3,5-dihydroxybenzoic acid^b^	4.21	153		
9	3-caffeoylquinic acid^b^	4.56	353		
10	ferulic acid-*O*-hexoside^b^	4.70			193
11	3,4-dihydroxybenzoic acid (PCA)^a^	5.00	153		
12	hydroxy-methoxybenzoic acid II^b^	5.34	167		
13	coumaric acid-*O*-hexoside^b^	5.53			163
14	sinapic acid-*O*-hexoside II^b^	5.96			223
15	2,5-dihydroxybenzoic acid (gentisic acid)^b^	6.32	153		
16	coumaroylquinic acid^b^	6.45		163	
17	sinapic acid-*O*-hexoside III^b^	6.62			223
18	5-caffeoylquinic acid (CQA)^a^	6.64	353		
19	hydroxy-dimethoxybenzoic acid II^b^	6.88	197		
20	trihydroxybenzoic acid I^b^	7.19	169		
21	2,6-dihydroxybenzoic acid^b^	7.24	153		
22	hydroxy-methoxybenzoic acid III^b^	7.25	167		
23	unknown	7.26	137		
24	4-caffeoylquinic acid^b^	7.33	353		
25	3-feruloylquinic acid^b^	7.47		193	
26	4-hydroxybenzoic acid (pHBA)^a^	7.72	137		
27	quercetin hexoside I^b^	8.74			301
28	4-hydroxy-3-methoxybenzoic acid (VA)^a^	9.03	167		
29	hydroxy-dimethoxybenzoic acid III^b^	9.12	197		
30	caffeic acid (CA)^a^	9.38	179		
31	4-hydroxy-3,5-dimethoxybenzoic acid (SyA)^a^	9.39	197		
32	quercetin hexoside II^b^	9.53			301
33	unknown	9.61	609		
34	hydroxy-dimethoxybenzoic acid IV^b^	10.71	197		
35	kampferol-3-*O*-hexoside^b^	11.31			285
36	4-feruloylquinic acid^b^	11.62		193	
37	trihydroxybenzoic acid II^b^	13.46	169		
38	*p*-coumaric acid (CuA)^a^	14.04	163		
39	unknown	14.41	223		
40	unknown	14.89	609		
41	unknown	15.22	609		
42	sinapic acid (SA)^a^	15.86	223		
43	ferulic acid (FA)^a^	16.05	193		
44	quercetin-3-*O*-rutinoside (Ru)^a^	16.67	609		
45	kampferol-3-*O*-rutinoside^b^	17.78			[M-308-H]^−^ = 285
46	unknown	17.78	301		
47	2-hydroxybenzoic acid (salycilic acid)^b^	19.39	137		
48	quercetin (Q)^a^	27.68	301		
49	kampferol (K)^a^	30.25	285		
50	isoxanthumol^b^	32.76	353		

a Identified by comparison with standard.

b Tentatively identified.

**Table 2:** Amounts (mg/L beer) of Free Phenolic Compounds Quantitated by HPLC-ESI-MS/MS in the Regular (PA100, PA95, and PA85) and in the Low Alcoholic (LA-PA100, LA-PA95, and LA-PA85) Craft Beers^a^

compound	PA100 (mg/L beer)	LA-PA100 (mg/L beer)	PA95 (mg/L beer)	LA-PA95 (mg/L beer)	PA85 (mg/L beer)	LA-PA85 (mg/L beer)
GA	0.13 ± 0.01^b^	0.12 ± 0.01*	0.11 ± 0.01^b^	0.08 ± 0.01*	0.12 ± 0.01a	0.11 ± 0.02
PCA	0.12 ± 0.01^b^	nd	0.31 ± 0.05^b^	0.44 ± 0.01	0.25 ± 0.01^a^	nd
CQA	<0.24^#^	<0.24^#^	<0.24^#^	<0.24^#^	<0.24^#^	<0.24^#^
pHBA	0.42 ± 0.02^b^	0.36 ± 0.02*	0.41 ± 0.01^b^	0.30 ± 0.03*	6.06 ± 0.06^a^	2.54 ± 0.25*
VA	1.04 ± 0.10^b^	1.13 ± 0.10	1.05 ± 0.10^b^	1.16 ± 0.10	0.77 ± 0.03^a^	0.84 ± 0.03
CA	nd	nd	0.11 ± 0.01^b^	nd	0.12 ± 0.02^a^	0.20 ± 0.02*
SyA	nq	nq	nq	nq	nq	nq
CuA	0.28 ± 0.04^a^	0.31 ± 0.03	0.42 ± 0.03^b^	0.43 ± 0.04	0.20 ± 0.02^a^	0.41 ± 0.02*
SA	0.78 ± 0.01^b^	0.62 ± 0.02*	0.77 ± 0.04^b^	0.72 ± 0.02	1.60 ± 0.01^a^	0.53 ± 0.02*
FA	1.16 ± 0.08^a^	1.27 ± 0.02	1.53 ± 0.10^b^	1.73 ± 0.10	0.75 ± 0.03^a^	1.28 ± 0.08*
Ru	0.65 ± 0.08^a^	0.46 ± 0.06	0.72 ± 0.10^a^	0.58 ± 0.07	0.68 ± 0.06^a^	<0.29^#^
Q	<0.23^#^	<0.23^#^	<0.23^#^	<0.23^#^	<0.23^#^	<0.23^#^
K	<0.06^#^	<0.06^#^	<0.06^#^	<0.06^#^	<0.06^#^	<0.06^#^
TA	4.58	4.27	5.32	5.44	10.55	5.91
TPC (GAE mg/L beer)	361.3 ± 10.2^a§^	386.5 ± 18.3^a^	454.5 ± 17.7^b§^	456.3 ± 14.0^b^	463.7 ± 14.5^b§^	450.5 ± 5.0^b^
AA (TE μmol/L beer)	747.9 ± 40^a§^	793.3 ± 10^a^	780.3 ± 15^a§^	819.7 ± 15^ab^	852.1 ± 15^b§^	800.3 ± 15^ab^

a TA: total amount (mg/L beer) of free phenolic compounds for each regular and low alcoholic beer. Mean values ± SD from triplicate analysis PA with different letters within rows are significantly different (*p* < 0.05),* LA-PA significantly different from the corresponding PA (*p* < 0.05),^#^ LOQ previously reported.^33^^§^TPC and AA of PA were previously published,^34^ and they were reported herein for a direct comparison with LA-PA; nd = not detected; nq = not quantitated.

#### Total Phenol Determination

The TPC was determined by the Folin–Ciocalteu (FC) assay,^38,39^ by using GA as the reference compound for the calibration curve. Results were given as GA equivalent (GAE) mg/L beer and are shown in Table 2.

#### Antioxidant Activity Determination

The AA was evaluated by the DPPH assay,^40^ using a Perkin Elmer, Lambda Bio 40 spectrophotometer. The percentage inhibition of remaining DPPH was calculated, according to the literature.^13^ Trolox (T) was used as the reference compound for the calibration curve, and results were reported as T equivalent (TE) μmol/L beer, as shown in Table 2.

#### ESI-MS/MS Infusion Experiments

Regular and low alcoholic beers were degassed, filtered, diluted 1:10 with the mobile phase, and analyzed by direct infusion into the ESI source.^33^ The samples were infused with 5 μL/min with an external syringe; spectral data were acquired for 2 min in the mass range 80–800 Da, in negative ionization (ES–, 28 V cone voltage), and in positive ionization (ES+, 24 V cone voltage).

#### NMR Experiments

The craft beers as received were degassed for 10 min in an ultrasonic bath. The samples for analysis were prepared directly in a 5 mm NMR tube by mixing 640 μL of beer with 160 μL of D_2_O containing 5 mM sodium azide and 5 mM of 3-(trimethylsilyl)-propionic-2,2,3,3-*d*_4_ acid sodium salt (TSP). NMR spectra were recorded at 28 °C on a Bruker AVANCE 600 spectrometer operating at a proton frequency of 600.13 MHz and equipped with a Bruker multinuclear *z*-gradient inverse probehead. The Bruker zgpr ^1^H NMR pulse sequence was used, with suppression of water signal during the last 2 s of relaxation delay (8.3 s) by applying a soft pulse. The ^1^H spectra were acquired by co-adding 128 transients using a 90° pulse, 32 K data points, and spectral width 7183 Hz (12 ppm). ^1^H spectra were referenced to the TSP signal (CH_3_, 0.00 ppm). Spectra were processed using exponential multiplication before Fourier transform (FT) with 0.3 Hz line-broadening and zero filling to 64 K points. Manual phase correction was followed by automatic baseline correction. The integration of selected signals was performed manually, and all the integrals were normalized to that one of TSP at 0.0 ppm set to 100. The signal assignment was carried out, as previously reported.^41^ The integral regions of selected signals in ^1^H NMR spectra of beer are reported in Table S3. The molar concentrations of identified metabolites were calculated using integrals and known concentrations of the standard (TSP).

### Statistical Analysis

Brewing and dealcoholization trials were carried out in triplicate. Data of each alcoholic and dealcoholized beer were reported as means of three samples. The comparison between the means of the three different samples was carried out using one-way analysis of variance. The significance of differences (*p* < 0.05) among samples was determined by the Tukey test.

## Results and Discussion

Three top-fermenting Pale Ale craft beers, named PA100, PA95, and PA85 according to the increasing percentage of dark malt used for brewing, had an ABV in the range 6.2–6.8 vol %. After four dealcoholization cycles by OD, an ABV slightly less than 1 vol % was obtained for all the beers. All the samples were tested for TPC and AA; the phenolic profile was analyzed by HPLC-ESI-MS/MS analysis; other metabolites profile was investigated by ESI-MS/MS infusion and NMR experiments. The phenolic profile and the other metabolites profile are discussed below, separately.

### Phenolic Profile by HPLC-ESI-MS/MS

13 free phenolic compounds were identified by HPLC-ESI-MS/MS analysis, by matching the selected mass signal in the SIR chromatogram and the chromatographic retention time (*t*_R_) with standard. Moreover, each SIR chromatogram evidenced the presence of isobaric peaks that could be ascribed to (a) isomeric compounds, (b) fragments of bonded forms, or (c) unknown compounds. Among these peaks, 30 compounds were tentatively identified, as described below, while seven peaks remained unknown. Chromatographic and spectral data are resumed in Table 1.

The SIR chromatogram of the selected ion [M – H]^−^ = 137 *m*/*z* (Figure S1) evidenced three peaks: **26** was identified with STD as pHBA; **47** was tentatively identified as 2-hydroxybenzoic acid (salicylic acid), based on the high elution time, in agreement with a decreased polarity due to the intramolecular H-bond, and congruent with the literature.^28^ The isomer mHBA was not detected; **23** remained unknown. The peak ratio **26**:**23** strongly increased from PA100 to PA85, suggesting a prevalence of pHBA in the Caraamber malt.

The SIR chromatogram of the selected ion [M – H]^−^ = 153 *m*/*z* (Figure S2) evidenced four peaks: **11** was identified with STD as PCA; **8**, **15**, and **21** were tentatively identified as the isomers 3,5-dihydroxybenzoic acid, 2,5-dihydroxybenzoic acid (gentisic acid), and 2,6-dihydroxybenzoic acid, respectively, based on a decreasing polarity order and in good agreement with the literature.^28,29^**11** and **21** were the most abundant ones; the percentage of **8** increased from PA100 to PA85.

The SIR chromatogram of the selected ion [M – H]^−^ = 169 *m*/*z* (Figure S3) evidenced three peaks: **2** was identified as GA with STD; **20** and **37** were tentatively assigned to isomeric forms with lower polarity likely due to the intramolecular H-bond. They are reported in Table 1 as trihydroxybenzoic acid I and II, respectively.

The SIR chromatogram of the selected ion [M – H]^−^ = 167 *m*/*z* (Figure S4) evidenced four main peaks: **28** was identified with STD as VA; **5**, **12**, and **22**, at lower *t*_R_, were tentatively assigned to isomeric compounds with higher polarity. No hydroxy-methoxybenzoic acids, except VA, were previously reported in beer, at least to the best of our knowledge. **5**, **12**, and **22** are reported in Table 1 as hydroxy-methoxybenzoic acid I, II, and III, respectively. Different abundance was found: VA was the most abundant peak in all the beers, while **5**, **12**, and **22** decreased in percentage from PA100 to PA85.

The SIR chromatogram of the selected ion [M – H]^−^ = 197 *m*/*z* (Figure S5) evidenced five main peaks: **31** was identified with STD as SyA; **4**, **19**, **29**, and **34** were tentatively assigned to isomeric compounds. **34** was in percentage the most abundant in all the samples: its high *t*_R_ compared to SyA suggested the structure of a 2-hydroxy-dimethoxybenzoic acid. Since no data for comparison were found in the literature, **4**, **19**, **29**, and **34** were named as hydroxy-dimethoxybenzoic acid I, II, III, and IV, respectively, and are listed in Table 1. Therefore, a variety of hydroxy-methoxybenzoic acids in beer was suggested by these data.

The SIR chromatogram of the selected ion [M – H]^−^ = 353 *m*/*z* (Figure S6) evidenced four peaks: **50** was tentatively identified as isoxanthohumol, based on the high *t*_R_ and in agreement with the literature.^29,30^**18** was identified with STD as CQA; **9** and **24** were tentatively assigned to 3-caffeoylquinic acid and 4-caffeoylquinic acid, respectively. Peaks **9** and **24** were prevalent, compared to **18**, in all the samples, in good agreement with the literature reporting 3-CQA and 4-CQA in beer.^30^ Moreover, the chromatographic elution order is in good agreement with the literature on CQAs.^29−33,42,43^ The SIR chromatogram of the selected ion [M – H]^−^ = 179 *m*/*z* (Figure S7) further supported those assignments. In fact, besides **30** identified as CA with STD, two peaks with the same *t*_R_ of **9** and **24** were observed: they were likely ascribed to the [M-174-H]^−^ = 179 *m*/*z* fragment due to the loss of the quinic acid moiety.^29,31^**9**, **24**, and **30** were not detected in P100 samples. The last isobaric peak **3**, eluted at low *t*_R_, was observed in all the beers: it was ascribed to the [M-162-H]^−^ = 179 *m*/*z* fragment of a caffeic acid-*O*-hexoside, due to the loss of an hexose moiety, in agreement with the literature.^31^ This assignment was also consistent with the decreasing percentage observed from PA100 to PA85, likely due to the Maillard reaction, favored at the higher temperature used for the production of caramel malts.^44^

The SIR chromatogram of the selected ion [M – H]^−^ = 163 *m*/*z* (Figure S8) evidenced three peaks: **38** was identified with STD as CuA; **13** and **16** were ascribed to fragments of compounds with higher polarity. On the basis of data previously reported,^33^**16** was tentatively identified as a coumaroylquinic acid, evidenced as the fragment [M-174-H]^−^ = 163 *m*/*z* due to the loss of the quinic acid moiety.^29^**13** was ascribed to the fragment [M-162-H]^−^ = 163 *m*/*z* and tentatively identified as a coumaric acid-*O*-hexoside, congruent with the literature.^31^

The SIR chromatogram of the selected ion [M – H]^−^ = 193 *m*/*z* (Figure S9) evidenced four main peaks: **43** was identified as FA with STD; **10**, **25**, and **36** were ascribed to fragments of polar derivatives. **25** and **36** were tentatively assigned to the fragment [M-174-H]^−^ = 193 *m*/*z* of 3-feruloylquinic acid and 4-feruloylquinic acid, respectively, whose presence in beer was recently reported.^29−31,45^ The elution order was in good agreement with the literature.^29,33^ 5-feruloylquinic acid was excluded because no fragment 193 *m*/*z* was reported for it in the literature.^29^**10** was ascribed to the fragment [M-162-H]^−^ = 193 *m*/*z* and tentatively identified as a ferulic acid-*O*-hexoside, which provides the fragment 193 *m*/*z*, according to the literature.^31^

The SIR chromatogram of the selected ion [M – H]^−^ = 223 *m*/*z* (Figure S10) evidenced five main peaks: **42** was identified as SA with STD. **7**, **14**, and **17** were likely due to fragments of high polarity compounds, whose percentage abundance compared to peak **42** decreased from PA100 to PA85. Since sinapic acid-*O*-hexosides were found in beer and the fragment [M-162-H]^−^ = 223 *m*/*z* was reported for them,^31^**7**, **14**, and **17** were tentatively assigned to three sinapic acid-*O*-hexosides, named I, II, and III, respectively, and are listed in Table 1. **39** remained unknown.

The SIR chromatogram of the selected ion [M – H]^−^ = 609 *m*/*z* (Figure S11) evidenced four main peaks: **44** was identified as quercetin-3-*O*-rutinoside (Rutin, Ru) with STD. **33**, **40**, and **41** were neither tentatively assigned, and they are reported as unknown in Table 1.

The SIR chromatogram of the selected ion [M – H]^−^ = 301 *m*/*z* (Figure S12) evidenced five peaks: **48** was assigned to Q with STD. **46** and the highly polar **1** are reported as unknown in Table 1. **27** and **32** were tentatively assigned to quercetin hexosides I and II, respectively: the elution order and the fragment [M-162-H]^−^ = 301 *m*/*z* were congruent with the literature.^29,31,46^

The SIR chromatogram of the selected ion [M – H]^−^ = 285 *m*/*z* (Figure S13) evidenced four main peaks: **49** was identified as K with STD. **35** was tentatively identified as a kampferol-3-*O*-hexoside, based on the *t*_R_ and the fragment [M-162-H]^−^ = 285 *m*/*z*, congruent with the literature.^29,31^**45** was tentatively assigned to kampferol-3-*O*-rutinoside, previously found in beer and providing the fragment [M-308-H]^−^ = 285 *m*/*z* due to the loss of the rutinose moiety.^29^ Moreover, the elution time was similar to that one of quercetin-3-*O*-rutinoside **44** (Ru). **6** is reported in Table 1 as unknown.

Noteworthy, the tentatively assigned quercetin-hexosides (**27** and **32**) and kampferol-hexoside (**35**) were in the same elution range (8.74–11.31 min), and their elution order was consistent with that one of Q and K. These comparison seemed to further support the assignments. Moreover, **1** and **6** (Figures S12 and S13, respectively) suggested the presence of similar highly polar derivatives of Q and K.

Summing up, a similar phenolic profile was observed for the three regular craft beers PA100, PA95, and PA85, except for CA that was not detected in PA100 while it was found in PA95 and PA85. Some significant difference observed in PA85 compared to PA100, mainly regarding the relative phenolic distribution, were likely due to the higher percentage of caramel malt in PA85. In fact, the thermal processing step is responsible of changes in the malt phenolic content because of chemical reactions as degradation, isomerization, polymerization, and Maillard reaction.^25,26,43^

A similar phenolic profile was obtained for the low alcoholic beers LA-PA100, LA-PA95, and LA-PA85. The ES− chromatograms, one for each selected mass, recorded for LA-PA85 are shown as an example in Figures S14–S16.

### Free Phenolic Compound Quantitation

The analytical method previously developed^33^ has been improved by adding the validation parameters for five STDs: GA, PCA, CA, SyA, and SA. A good linearity was found in the range 10–100 μg/L, as evidenced by *R*^2^ values in the range 0.9815–0.9990 (see Table S1), the calibration data being satisfactory using the calibration equation after plotting and examining the regression statistics, with a 95% confidence level. LOD and LOQ values were found in the range 10–40 μg/L (1.39–5.05 pmol injected) and 30–120 μg/L (4.16–15.22 pmol injected), respectively (see Table S1). Satisfactory results were obtained for interday precision, mean value 3.38% (range 1.07–6.19%), and intraday precision, mean value 4.11% (range 3.48–5.08%), accuracy, ranging from −22 to 10% (see Table S2). These results were well aligned with those ones of the other STDs (CQA, pHBA, VA, mHBA, CuA, FA, Ru, Q, and K) previously reported^33^ and consistent with the literature.^28^ Recovery was found in the range 91.98–115.03%, with RSD % in the range 5.68–9.96% (see Table S2). ME was found in the range −16.02 to 26.27% (see Table S2). Such a ME from weak to medium^47^ confirmed the suitability of the method for the analysis of only diluted real samples.

For the sake of completeness, ME of FA, CuA, pHBA, VA, and Ru, previously reported for 1:100 diluted samples,^33^ was calculated in the 1:10 diluted samples, too. Only a slight worsening was observed: −20.34, 22.72, 21.89, 11.45, and 8.56% (not reported in Table S2), respectively, versus −23.79, −10.79, −4.59, 18.78, and 4.53%,^33^ respectively, which confirmed a weak to medium ME for all the investigated compounds.

The content of the phenolic compounds identified in PA and LA-PA beers is reported as mg/L beer in Table 2.

SyA (**31**) was identified but not quantified because of the co-elution with the isobaric **29** (Figure S5); CQA (**18**), Q (**48**), and K (**49**) resulted under LOQ.^33^

As a general trend, PA85 was significantly different (*p* < 0.05) compared to PA100, as expected because both the quantitative and qualitative phenolic profile depend on raw material, as reported in the literature.^23,33,48^ Adding the amounts of the quantitated phenolic compounds (TA, Table 2), PA85 content was around twice that one of PA100 (10.55 mg/L vs 4.58 mg/L), the increment mainly due to pHBA and SA. Ru was the most abundant flavonol, equally distributed in PA100, PA95, and PA85.

The identified phenolic compound contents in LA-PA beers were found to be different (PCA, VA, and Ru) or significantly different (*p* < 0.05, GA, pHBA, CA, CuA, SA, and FA) from those ones in the corresponding PA beers: as a general trend, decreasing values were observed (GA, PCA, pHBA, SA, and Ru, Table 2), but increasing values were also found (CuA, FA, VA, and CA, Table 2). Two main effects might cause these opposite trends: the loss of small-sized molecules through the membrane and a concentration increasing due to the loss of ethanol and other alcohols (see Table 3). Hence, the amount of each compound in LA-PA beers likely depends on these combined effects: a prevalent concentration effect combined with a weak or no loss might occur for CuA, FA, VA, and CA, while a major loss might occur for the other compounds, especially for pHBA and SA. In fact, the major loss was observed for LA-PA85 (−44%, TA, Table 2), in which the slight increase of CuA, FA, VA, and CA only weakly counteracts the strong decrease of pHBA and SA.

**Table 3:** Amounts of Metabolites (mmol/L) Quantitated by NMR in the Regular (PA100, PA95, and PA85) Craft Beers and Loss % in the Low Alcoholic (LA-PA100, LA-PA95, and LA-PA) Craft Beers^a^

metabolite	PA100 (mmol/L)	PA95 (mmol/L)	PA85 (mmol/L)	LA-PA100 (loss %)	LA-PA95 (Loss %)	LA-PA85 (Loss %)
EtOH	1120.3 ± 50.0^a^	1154.8 ± 2.3^a^	1165.8 ± 31.5^a^	80	90	88
isobutanol	0.61 ± 0.01^a^	0.68 ± 0.01^b^	0.74 ± 0.02^b^	84	85	81
isopentanol	0.75 ± 0.01^a^	0.90 ± 0.04^b^	0.88 ± 0.03^b^	77	88	84
propanol	0.49 ± 0.02^a^	0.49 ± 0.03^a^	0.50 ± 0.05^a^	77	88	89
2-phenylethanol	0.42 ± 0.006^a^	0.48 ± 0.004^a^	0.52 ± 0.040^a^	27	40	59
Amino Acids						
alanine	0.15 ± 0.01^a^	0.15 ± 0.01^a^	0.30 ± 0.004^b^	72	62	83
proline	3.66 ± 0.14^a^	3.17 ± 0.06^b^	3.35 ± 0.04 ^ab^	19	15	36
pyroglutamate	1.58 ± 0.04^a^	1.62 ± 0.02^a^	1.61 ± 0.09^a^	30	21	26
tyrosine	0.32 ± 0.02^a^	0.32 ± 0.01^a^	0.35 ± 0.03^a^	30	32	53
histidine	0.19 ± 0.01^a^	0.20 ± 0.01^a^	0.21 ± 0.01^a^	nd	nd	nd
GABA	nq	nq	nq			
phenylalanine	nq	nq	nq			
valine	nq	nq	nq			
Organic Acids						
acetic A	1.47 ± 0.02 ^ab^	1.40 ± 0.02^a^	1.60 ± 0.05^b^	52	48	61
lacticA	4.85 ± 0.10^a^	4.21 ± 0.004^b^	4.33 ± 0.03^b^	9	11	–25
pyruvic A	0.86 ± 0.03^a^	0.80 ± 0.003^ab^	0.74 ± 0.01^b^	0	0	–42
succinic A	2.63 ± 0.09^a^	2.94 ± 0.03^b^	3.12 ± 0.09^b^	0	0	30
fumaric A	0.038 ± 0.002^a^	0.03 ± 0.002^a^	0.03 ± 0.01^a^	28	21	0
gallic A	0.15 ± 0.01^a^	0.16 ± 0.004^a^	0.17 ± 0.01^a^	0	0	22
citric A	nq	nq	nq			
Carbohydrates						
maltodextrines α(1–6)	25.80 ± 0.74^a^	28.43 ± 0.03^a^	28.87 ± 1.68^a^	0	0	21
reduced end units	15.00 ± 0.45^a^	18.12 ± 0.08^b^	17.76 ± 0.75^b^	18	13	0
maltodextrines α(1–4)	171.12 ± 7.11^a^	203.63 ± 0.32^b^	200.26 ± 9.16^b^	0	0	–15
Nucleosides						
adenosine	0.26 ± 0.01^a^	0.30 ± 0.01^b^	0.31 ± 0.01^b^	nd	nd	nd
cytidine	0.35 ± 0.03^a^	0.29 ± 0.04^a^	0.25 ± 0.004^a^	0	0	28
uridine	0.32 ± 0.01^a^	0.30 ± 0.02^a^	0.37 ± 0.03^a^	0	0	58
Miscellaneous						
choline	0.43 ± 0.02^a^	0.38 ± 0.002^a^	0.40 ± 0.01^a^	0	0	–14
glycerol	22.88 ± 0.66^a^	23.94 ± 0.14^a^	25.02 ± 0.70^a^	0	0	20
glycinebetaine	nq	nq	nq			
GP-choline	0.93 ± 0.04^a^	1.10 ± 0.01^b^	1.21 ± 0.02^b^	0	8	58

a Values with different letters^a,b^ within rows are significantly different (*p* < 0.05); values with ^ab^ letters are not significantly different with respect to^a^ and^b^ rows* significant (*p* < 0.05) loss % (LA-PA vs PA); nd = not detected; nq = not quantified.

Summing up, except for pHBA and SA in PA85, the dealcoholization process seemed not to have a strong effect on the content of free phenolic compounds, despite that were expected easier to be lost during the membrane process because of their small size.

A similar trend was observed for the TPC, as shown in Table 2: PA95 and PA85 were significantly different (*p* < 0.05) from PA100, with values increasing from PA100 to PA85. PA beers had a TPC in the range 361.3–463.7 mg GAE/L beer, in good agreement with values reported for commercial beers (152-486 mg GAE/L depending on the beer style).^49^ Similar TPC values were found in LA-PA beers, in the range 386.5–456.3 mg GAE/L beer.

The AA of PA and LA-PA beers was evaluated by the DPPH assay, and results are shown in Table 2. The AA strongly depends on the phenolic content. In fact, significantly increasing AA was found to be depending on the increasing percentage of colored malt. PA85 was significantly different (852.1 TE μmol/L beer) from PA100 (747.9 TE μmol/L beer) and PA95 (780.3 TE μmol/L beer). These data were in good agreement with those ones reported in the literature for commercial beers.^50^ No significant differences were found after dealcoholization.

All these data supported the suitability of the OD process to obtain low alcoholic beer with a low impact on taste and benefits.

### ESI-MS/MS Metabolic Profile

**Figure 1:**
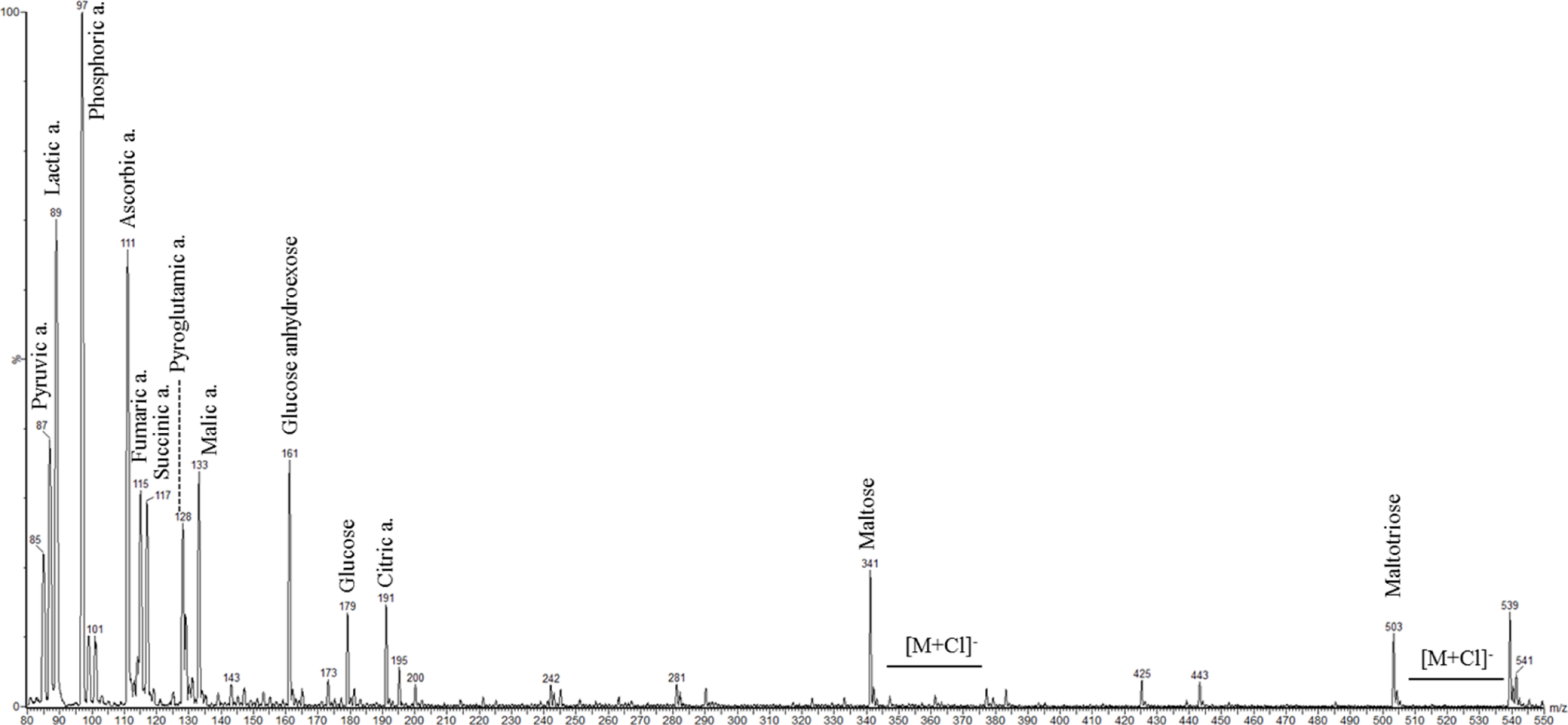
Negative electrospray ionization (ES−) mass spectrometry fingerprinting of PA85.

**Figure 2:**
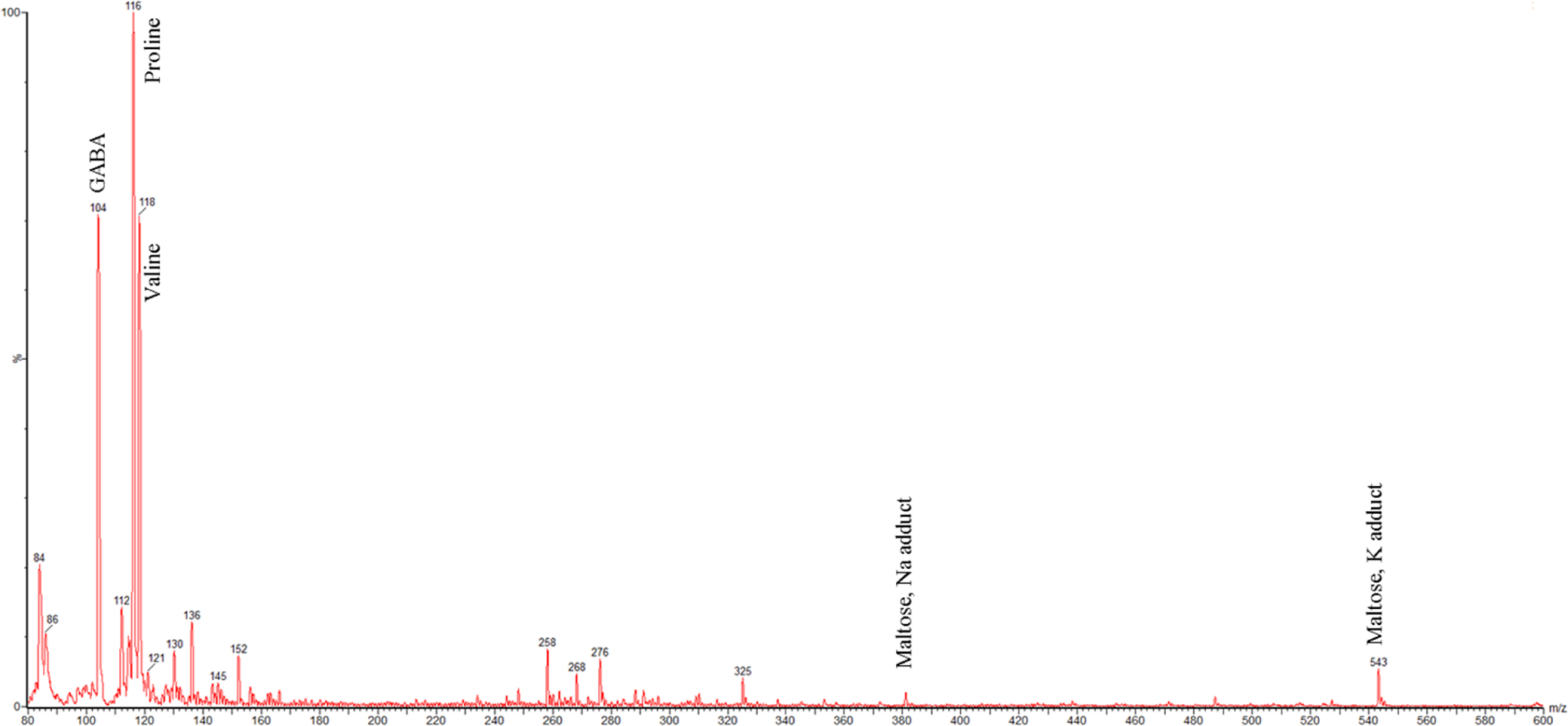
Positive electrospray ionization (ES+) mass spectrometry fingerprinting of PA85.

Electrospray ionization mass spectrometry fingerprinting of PA and LA-PA beers was obtained by direct infusion of the samples into the source. ES– and ES+ images of PA85 are shown as an example in Figures 1 and 2, respectively. The same metabolic profile was evidenced for PA100, PA95, and PA85, with little differences regarding the relative amounts (the signal height is referred to the higher one). None of the identified or tentatively identified phenolic compounds were therein evidenced, as expected because of their very low amount compared to that one of the other components as sugars or organic acids. The main signals were tentatively assigned by comparing their *m*/*z* values with data reported in the literature^5,33,51,52^ and NMR data, as shown in Table S3.

Characteristic ions in the *m*/*z* range 330–550 were assigned to oligosaccharides.

The anion [M – H]^−^ = 341 *m*/*z* (ES–, Figure 1) was assigned to maltose, supported by the chloride adduct [M + Cl]^−^ = 377 *m*/*z* (ES–, Figure 1) and the potassium adduct [M + K]^+^ = 381 *m*/*z* (ES+, Figure 2).

The anion [M – H]^−^ = 503 *m*/*z* (ES–, Figure 1) was assigned to maltotriose, supported by the chloride adduct [M + Cl]^−^ = 539 *m*/*z* (ES–, Figure 1), the sodium adduct [M + Na]^+^ = 527 *m*/*z* (ES+, Figure 2) and the potassium adduct [M + K]^+^ = 543 *m*/*z* (ES+, Figure 2).

The anion [M – H]^−^ = 179 *m*/*z* was assigned to the deprotonated glucose, and the anion [M – H]^−^ = 161 *m*/*z* was assigned to the deprotonated anhydrohexose of glucose (ES–, Figure 1), in agreement with the literature.^51^

Characteristic anions in the *m*/*z* range 80–200 were assigned to small organic acids, based on (i) the absence of the corresponding cations in the ES+ chromatograms, (ii) the loss of signal at high cone voltage values, with the characteristic loss −CO_2_ of 44 Da, and (iii) in agreement with NMR results (Table S3).

The anions [M – H]^−^ with *m*/*z* 87, 89, 115, 117, and 128 (ES–, Figure 1) were ascribed to pyruvic acid, lactic acid, fumaric acid, succinic acid, and pyroglutamic acid, respectively, and they were detected by NMR, too. Moreover, the anions [M – H]^−^ with *m*/*z* 97, 111, 133, and 191 (ES–, Figure 1) were ascribed to phosphoric acid, ascorbic acid, malic acid, and citric acid, respectively.

Characteristic cations evidenced in the range *m*/*z* 80–160 were assigned to amino acids, in agreement with the literature^33^ and by comparison with NMR data (Table S3).

The cations [M + H]^+^ with *m*/*z* 104, 116, and 118 (ES+, Figure 2) were ascribed to γ-aminobutyric acid (GABA), proline, and valine, respectively.

**Figure 3:**
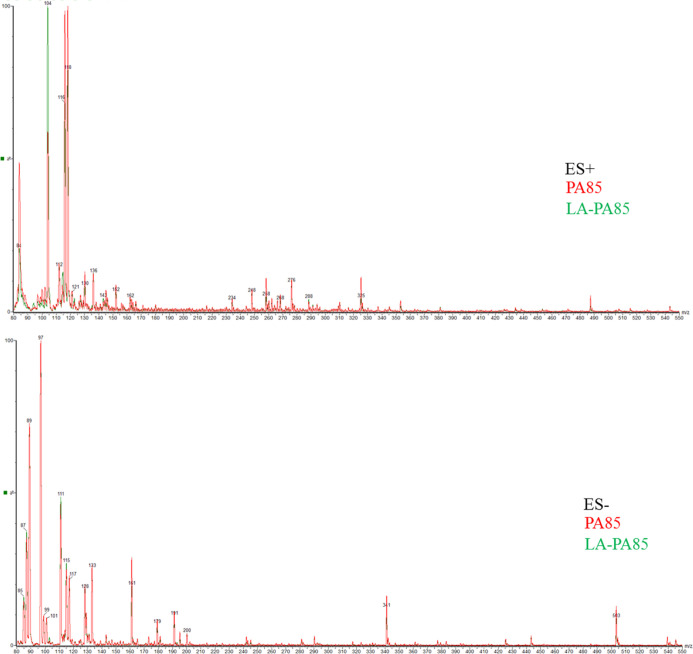
Superimposed ES– and ES+ metabolic profiles of PA85 (red chromatograms) and LA-PA85 (green chromatograms).

Little differences were evidenced in the metabolic profile of LA-PA beers compared with that one of PA beers. The superimposed images of PA85 (red line) and LA-PA85 (green line) in both ES– and ES+ are shown as an example in Figure 3. A decrease was observed for sugars, regarding mainly the anhydrohexose of glucose (161 *m*/*z*), glucose (179 *m*/*z*), and maltose (341 *m*/*z*) (ES–, Figure 3); no significant change was observed for the organic acids (ES–, Figure 3); an increase was observed for GABA (104 *m*/*z*), while a slight decrease was observed for proline (116 *m*/*z*) and valine (118 *m*/*z*) (ES+, Figure 3).

### NMR Metabolic Profile

Regular and low alcoholic beers were degassed and diluted by deuterated water (beer/D_2_O 4:1 v/v) before ^1^H NMR analysis. The assignment of NMR signals (reported in Table S3 and shown in Figure S17) relied on previous data^41^ and literature. Only the most abundant metabolites were identified and quantified, due to the limited sensitivity of NMR spectroscopy; most of them belong to the chemical classes of alcohols, carbohydrates, organic acids, amino acids, and nucleosides.

Three diagnostic signals were chosen for the quantitation of different key structural motifs of oligosaccharides because of the complexity of the beer carbohydrate fraction in which malto-oligosaccharides and limit dextrins are the main components deriving from starch hydrolysis.^53^ The linear α-glucose chains with α(1–4) glycosidic linkages were represented by the sum of signals in the 5.29–5.40 ppm range ascribed to anomeric CH-1 of α-glucopyranose rings. The branch points with α(1–6) glycosidic linkages were taken into account using the α-glucopyranose anomeric signal at 4.95 ppm. Finally, the reducing end α-glucose signal at 5.22 ppm was also quantitated.

Noteworthy, the monomeric α-glucose anomeric signal occupies the same place in the spectrum (5.23–5.22 ppm); therefore, additional data were necessary to confirm the assignment. All the other NMR signals of monomeric glucose were overlapped with those from malto-oligosaccharides, except the signal at 3.23 ppm (verified by the standard addition) that belong to the CH-2 group of β-glucopyranose. Taking into account that α- and β-anomeric forms of glucose were in equilibrium, and that only traces of 3.23 ppm signal were observed in the ^1^H spectrum of all the beer samples, the contribution of monomeric α-glucose anomeric signal could be neglected.

Most of the identified metabolites were quantitated, except a few ones whose concentrations were too low (GABA, phenylalanine, and valine), or whose signals were too large (citric acid) and/or partially overlapped with the signals of other components (glycinebetaine).

### NMR Metabolite Quantitation

The difference in the malt composition of PA100, PA95, and PA85 was reflected in their metabolic profile. Results are shown in Table 3.

A slightly lower content of reduced end glucose units and linear chain glucose α(1–4) units was observed in PA100 compared to PA95 and PA85. Noteworthy, the ratio of reduced end units to α(1–4) ones remains constant in all the beer samples indicating that medium-chain length remained probably constant, notwithstanding the variation of the oligosaccharide concentration.

A lower content of isobutanol, isopentanol, 2-phenylethanol, succinic acid, glycerophosphocholine, and adenosine was observed in PA100 compared to PA95 and PA85, too.

Conversely, a relatively higher content of proline, lactic acid, and pyruvic acid was observed in PA100 compared to PA95 and PA85.

A quite similar composition was observed for PA95 and PA85, except a few differences. PA85 showed the highest concentration of alanine and acetic acid and the lowest content of pyruvic acid, whereas the lowest content of proline was observed in PA95.

Metabolites in LA-PA beers are reported in Table 3 as a significant (*p* < 0.05) loss in percentage compared to PA beers.

The concentration of ethanol and other alcohols (isopentanol, isobutanol, and propanol) dropped from 5 to 10 times in LA-PA beers, whereas a content loss up to 60% was observed for 2-phenylethanol. These data are in agreement with a previous work.^12^ The lowest ethanol content was found in LA-PA95 beer (one-tenth that of PA95).

A significant content decrease was also observed for alanine, proline, pyroglutamate, and tyrosine. In particular, LA-PA85 showed the highest drop in the content of amino acids with respect to PA85. The highest decrease in LA-PA beers was observed for alanine. Histidine was not quantified in LA-PA beers due to the widening of its characteristic signal at 7.99 ppm, with a consequent signal-to-noise ratio too low for quantitation.

Not uniform changes were observed regarding the organic acid content: the acetic acid content dropped in all LA-PA beers, whereas the content of lactic acid and fumaric acid was slightly lower only in LA-PA100 and LA-PA95. In the case of LA-PA85, the pattern of changes in the organic acid content was quite specific, with a content increase for lactic acid and pyruvic acid and a content decrease for succinic acid and GA.

A loss from 13 to 18% of reduced end units in the carbohydrate fraction was observed in LA-PA100 and LA-PA95; an increase of the linear chain glucose α(1–4) unit content and a decrease of the branch point α(1–6) glucose content were observed in LA-PA85.

LA-PA85 showed a particular pattern of changes also in the case of glycerophosphocholine, glycerol, cytidine, and uridine, whose content dropped compared to PA85, whereas the content of choline increased. No changes were observed in LA-PA100 and LA-PA95 regarding the content of these metabolites, except glycerophosphocholine whose content slightly dropped in LA-PA95.

## Supporting Information Available

Correlation coefficient *R*^2^ values, linear equations of the calibration curves, LOD and LOQ of GA, protocatechuic acid (PCA), CA, SyA, and SA (25 μL injected, in triplicate analysis); interday and intraday precision (RSD %), accuracy (%), recovery (%), ME of GA, PCA, CA, SyA, and SA; integral regions of selected signals in ^1^H NMR spectra of the analyzed craft beers; ES– channel of the anion [M – H]^−^ = 137, 153, 169, 167, 197, 353, 179, 163, 193, 223, 609, 301, and 285 *m*/*z* in PA100, PA95, and PA85 and in STD solution for comparison, from bottom to top, respectively, identified with STD, tentatively assigned; ES– channel for GA (*m*/*z* 169), PCA (*m*/*z* 153), pHBA (*m*/*z* 137), VA (*m*/*z* 167), CA (*m*/*z* 179), and SyA (*m*/*z* 197), from bottom to top, in 1:10 diluted LA-PA85; ES– channel for CuA (*m*/*z* 163), SA (*m*/*z* 223), FA (*m*/*z* 193), and Ru (*m*/*z* 609), in 1:10 diluted LA-PA85; ES– channel for CQA (*m*/*z* 353), Q (*m*/*z* 301), and K (*m*/*z* 285), in 1:10 diluted LA-PA85; 600 MHz ^1^H NMR spectrum of PA100; and assignments: **1**, lactic acid; **2**, iso-pentanol; **3**, alanine; **4**, propanol; **5**, iso-butanol; **6**, proline; **7**, acetic acid; **8**, pyruvic acid; **9**, pyroglutamic acid; **10**, succinic acid; **11**, 2-phenylethanol; **12**, choline; **13**, glycerophosphocholine; **14**, glycerol; **15**, maltodextrines α-glucose (1–6) units; **16**, maltodextrines α-glucose (reduced end) units; **17**, maltodextrines α-glucose (1–4) units; **18**, fumaric acid; **19**, tyrosine; **20**, GA; **21**, uridine; **22**, cytidine; **23**, histidine; and **24**, adenosine (PDF)
