# Supplementary material for: Impact of Dealcoholization by Osmotic Distillation on Metabolic Profile, Phenolic Content, and Antioxidant Capacity of Low Alcoholic Craft Beers with Different Malt Compositions
Source: J Agric Food Chem. 2021 Apr 15;69(16):4816–26. doi: 10.1021/acs.jafc.1c00679 (PMC8154560; doi:10.1021/acs.jafc.1c00679)

SUPPORTING INFORMATION

Impact of Dealcoholization by Osmotic Distillation on Metabolic Profile, Phenolic Content, and Antioxidant Capacity of Low Alcoholic Craft Beers with Different Malt Compositions

Rita Petrucci,[a*] Paola Di Matteo,[b] Anatoly P. Sobolev,[c] Loredana Liguori,[d] Donatella Albanese,[d] Noemi Proietti,[c] Martina Bortolami,[a] and Paola Russo [b]

[a] *Department of Basic and Applied Sciences for Engineering, Sapienza University of Rome, Via del Castro Laurenziano 7, 00161 Rome, Italy*

[b] *Department of Chemical Engineering Materials Environment, Sapienza University of Rome, Via Eudossiana 18, 00184 Rome, Italy*

[c] *"Segre-Capitani" Magnetic Resonance Laboratory, Institute for Biological Systems, National Research Council (CNR), via Salaria km 29.300, 00015 Monterotondo, Rome, Italy*

[d] *Department of Industrial Engineering, University of Salerno, Via Giovanni Paolo II 132, 84084 Fisciano, SA, Italy*

*Corresponding author at: Department of Basic and Applied Sciences for Engineering, Sapienza University of Rome, Via del Castro Laurenziano 7, 00161 Rome, Italy – rita.petrucci@uniroma1.it

E-mail addresses: rita.petrucci@uniroma1.it (R.P.) ORCID 0000-0003-2411-825X; p.dimatteo@uniroma1.it (P.D.M.); martina.bortolami@uniroma1.it (M.B.); dalbanese@unisa.it (D.A.); lliguori@unisa.it (L.L.); anatoly.sobolev@cnr.it (A.P.S.) ORCID 0000-0001-8709-7666; noemi.proietti@cnr.it (N.P.); paola.russo@uniroma1.it (P.R.) ORCID 0000-0001-6877-6356

Index

Table S1……………………………………………………………………p. 3

Table S2……………………………………………………………………p. 4

Table S3……………………………………………………………………p. 5

Figure S1…………………………………………………………………p. 6

Figure S2…………………………………………………………………p. 7

Figure S3…………………………………………………………………p. 8

Figure S4…………………………………………………………………p. 9

Figure S5…………………………………………………………………p. 10

Figure S6…………………………………………………………………p. 11

Figure S7…………………………………………………………………p. 12

Figure S8…………………………………………………………………p. 13

Figure S9…………………………………………………………………p. 14

Figure S10………………………………………………………………..p. 15

Figure S11………………………………………………………………..p. 16

Figure S12……………………………………………………………..…p. 17

Figure S13…..p. 18

Figure S14……………………………………………………………..…p. 19

Figure S15……………………………………………………………..…p. 20

Figure S16……………………………………………………………..…p. 21

Figure S17……………………………………………………………..…p. 22

Table S1. Correlation coefficient R^2 values, linear equations of the calibration curves, LOD and LOQ of GA, protocatechuic acid (PCA), CA, SyA, and SA (25µL injected, in triplicate analysis).

STD	R^2	LOD (µg/L)	LOD (pmol/inj)	LOQ (µg/L)	LOQ (pmol/inj)	Linear equation
GA	0.9918	20	2.94	50	7.35	$y=1.54\text{x}10^5\ x - 3.89\text{x}10^2$
PCA	0.9815	10	1.62	30	4.87	$y=2.40\text{x}10^5\ + 7.75\text{x}10^2$
CA	0.9990	10	1.39	30	4.16	$y=3.99\text{x}10^5\ x + 1.60\text{x}10^3$
SyA	0.9947	40	5.05	120	15.22	$y=6.01\text{x}10^4\ x + 1.00\text{x}10^2$
SA	0.9903	30	3.34	90	10.04	$y=1.09\text{x}10^5\ x - 1.57\text{x}10^2$

Table S2. Interday and intraday precision (RSD %), accuracy (%), recovery (%), ME of GA, PCA, CA, SyA, and SA.

STD	Inter-day[a] (RSD %)	Intra-day[b] (RSD %)	Accuracy[c] (%)	Recovery[d] (%)	Recovery RSD %	ME[d]
GA	6.19	5.08	-8	99.04	5.68	26.27
	3.64			95.01		
	2.80			103.26		
				96.85		
				109.33		
PCA	3.87	4.52	4	95.24	6.99	23.07
	3.67			100.88		
	2.45			112.55		
				96.44		
				110.75		
CA	3.72	3.48	-6	96.70	6.47	-16.02
	4.89			92.16		
	1.86			94.33		
				92.82		
				107.43		
SyA	2.22	3.81	-22	97.37	5.79	24.09
	5.60			107.38		
	1.07			103.25		
				109.39		
				95.92		
SA	5.03	3.67	10	108.48	9.96	-8.16
	1.82			105.23		
	1.86			92.16		
				91.98		
				115.03		

[a] triplicate analysis in three different days (14, 50, 100 µg/L); [b] five injections (50 µg/L); [c] triplicate analysis (25 µg/L); [d] five spikes (10, 14, 20, 50, 100 µg/L) in duplicate analysis.

Table S3. Integral regions of selected signals in ^{1}H NMR spectra of the analyzed craft beers.

	**Compound**	**Group**	**Range (F1) from**	**Range (F1) to**
	TSP	$Si(CH_3)_3$	0.109	-0.111
	Ethanol	CH_3	1.249	1.095
**1**	Lactic Acid	CH_3	1.355	1.316
**2**	Iso-Pentanol	CH_2	1.45	1.406
**3**	Alanine	CH_3	1.466	1.452
**4**	Propanol	CH_2	1.56	1.51
**5**	Iso-Butanol	CH	1.749	1.725
**6**	Proline	CH_2	2.004	1.949
**7**	Acetic Acid	CH_3	2.022	2.013
**8**	Pyruvic Acid	CH_3	2.368	2.345
**9**	Pyroglutamic acid	CH	2.531	2.455
**10**	Succinic Acid	$(CH_2)_2$	2.573	2.542
**11**	2-Phenylethanol	CH_2	2.874	2.835
**12**	Choline	$N(CH_3)_3$	3.195	3.174
**13**	Glycerophosphocholine	$N(CH_3)_3$	3.224	3.201
**14**	Glycerol	CH	3.556	3.537
**15**	α-Glucose (1-6) units	CH	4.988	4.910
**16**	α-Glucose (reduced end) units	CH	5.249	5.188
**17**	α-Glucose (1-4) units	CH	5.444	5.253
**18**	Fumaric Acid	$(CH)_2$	6.58	6.568
**19**	Tyrosine	CH	6.904	6.868
**20**	Gallic Acid	CH	7.056	7.027
**21**	Uridine	CH	7.875	7.844
**22**	Cytidine	CH	7.936	7.905
**23**	Histidine	CH	8.004	7.986
**24**	Adenosine	CH	8.273	8.251

Figure S1. ES- channel of the anion $[M-H]^-$ = 137 *m/z* in PA100, PA95, PA85 and in STD solution for comparison, from bottom to top, respectively, [a] identified with STD, [b] tentatively assigned.

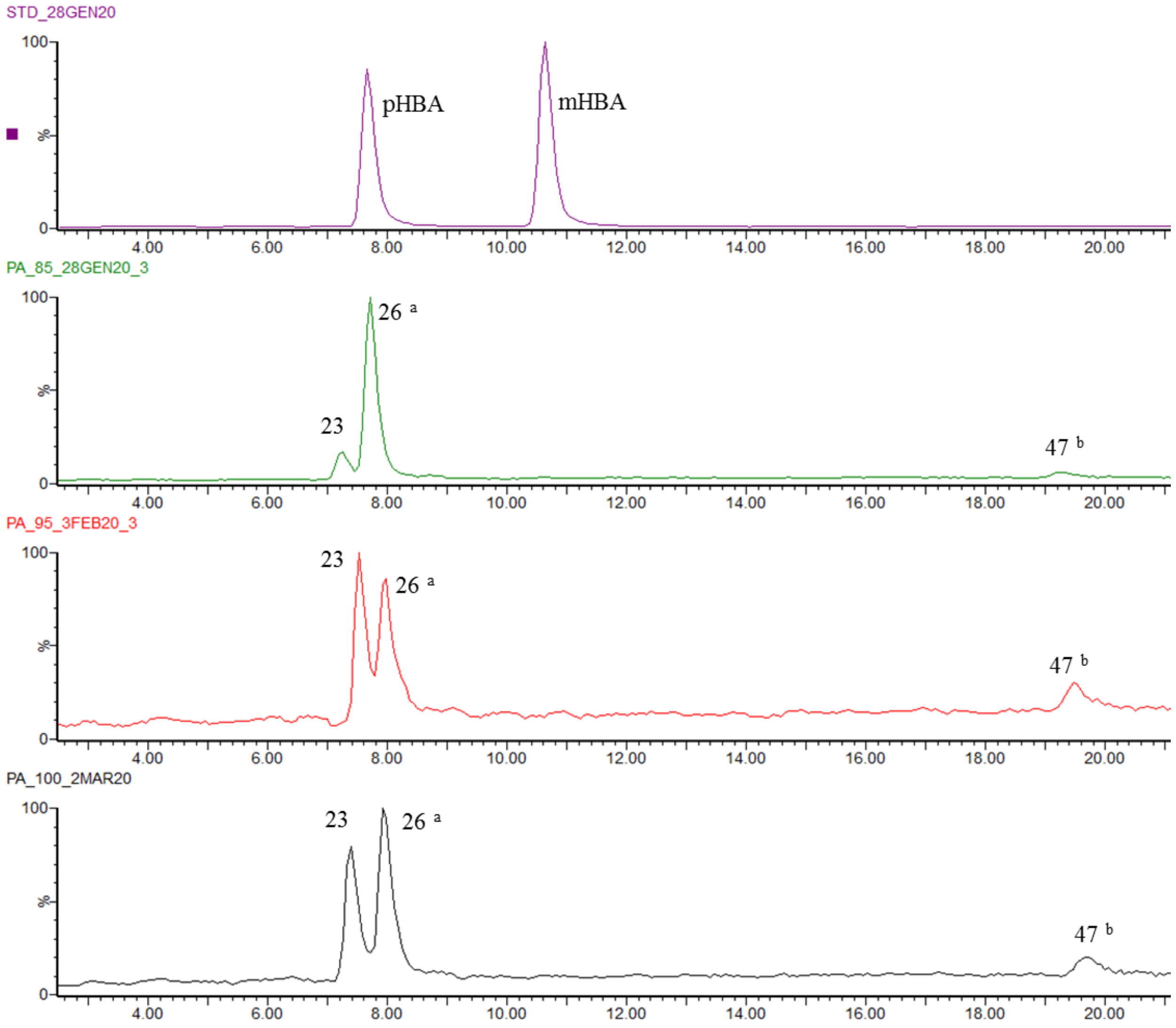

Figure S2. ES- channel of the anion $[M-H]^-$ = 153 *m/z* in PA100, PA95, PA85 and in STD solution for comparison, from bottom to top, respectively, [a] identified with STD, [b] tentatively assigned.

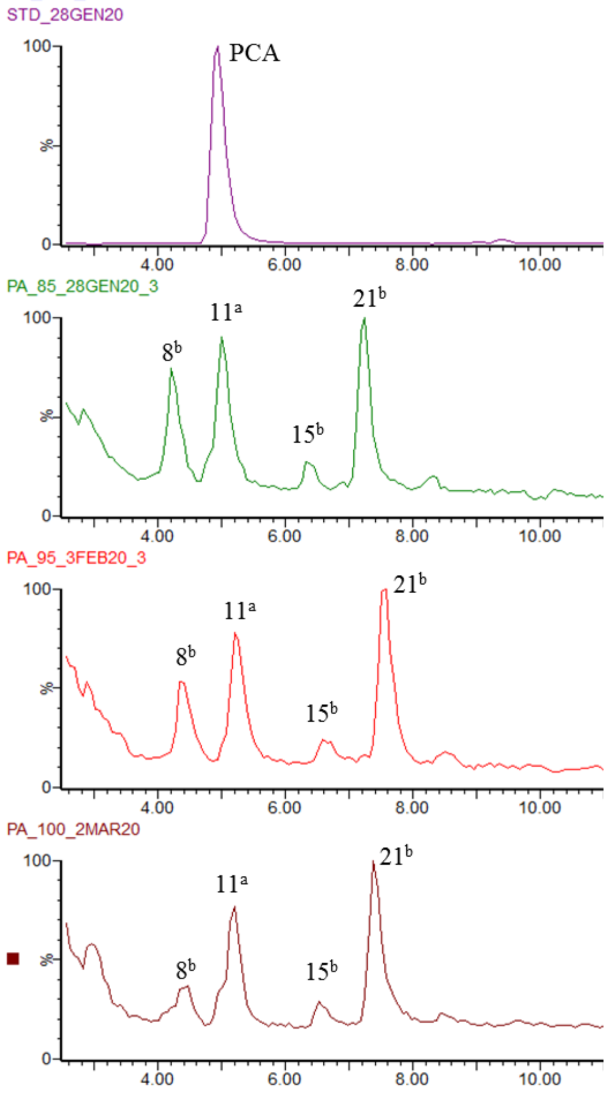

Figure S3. ES- channel of the anion $[M-H]^-$ = 169 *m/z* in PA100, PA95, PA85 and in STD solution for comparison, from bottom to top, respectively, [a] identified with STD, [b] tentatively assigned.

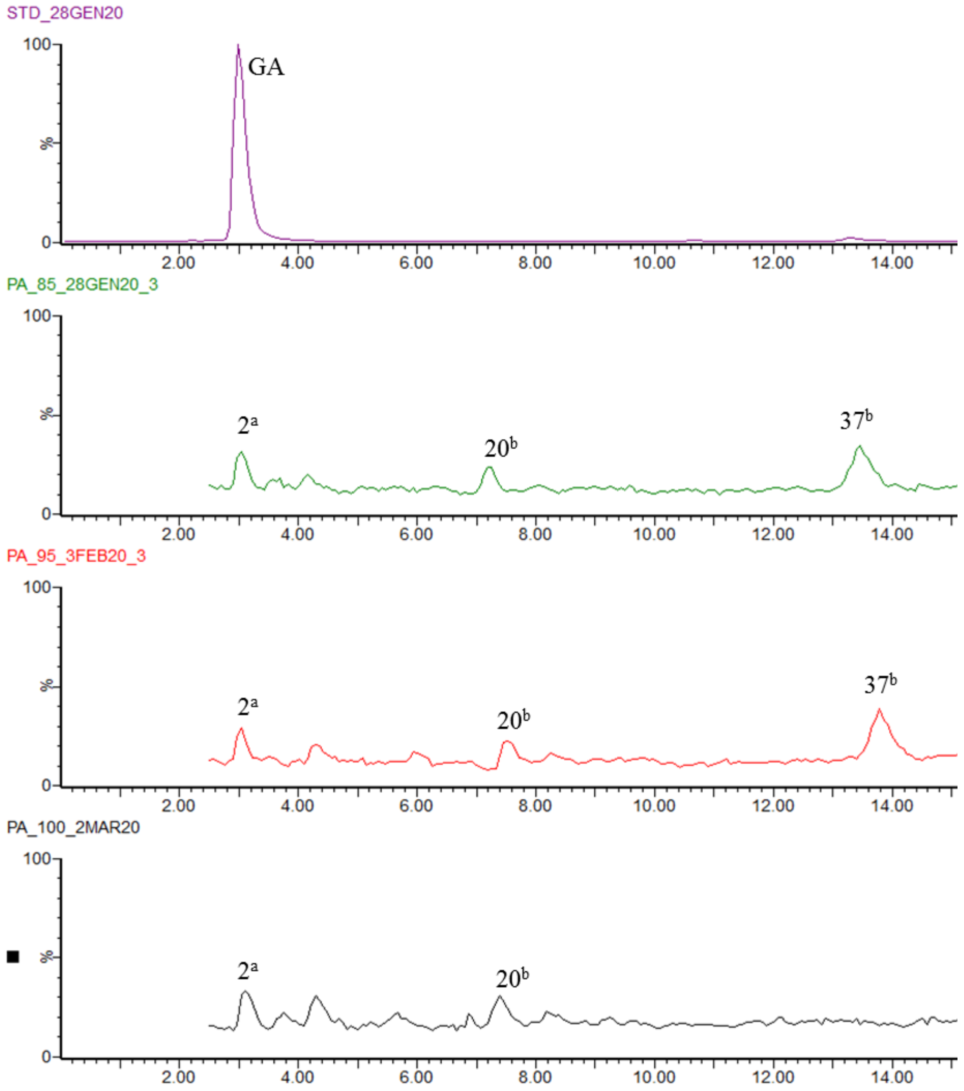

Figure S4. ES- channel of the anion $[M-H]^-$ = 167 *m/z* in PA100, PA95, PA85 and in STD solution for comparison, from bottom to top, respectively, [a] identified with STD, [b] tentatively assigned.

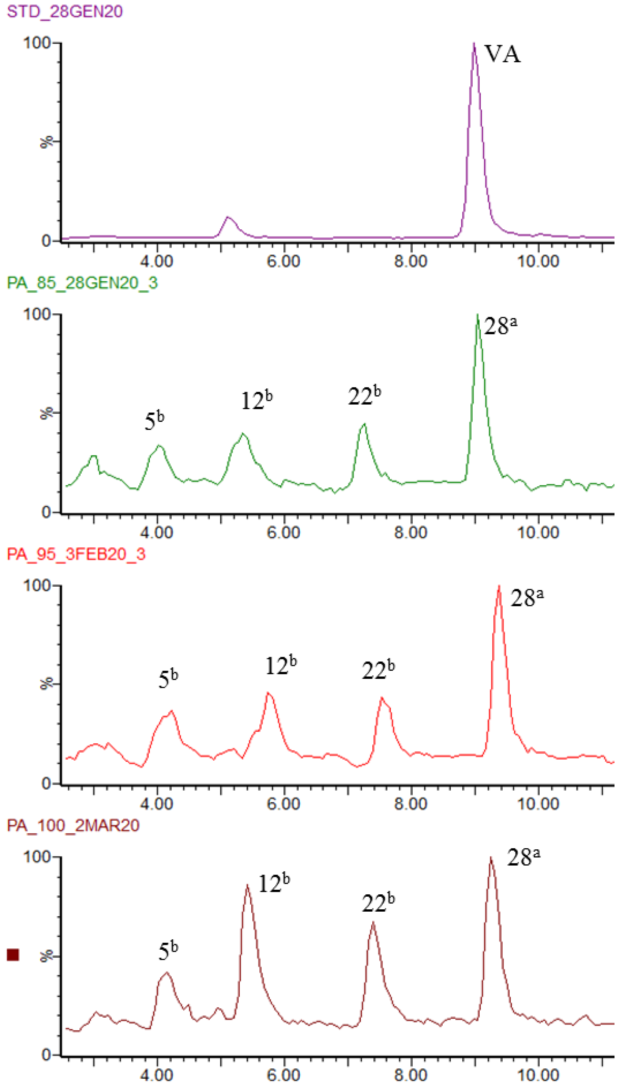

Figure S5. ES- channel of the anion $[M\text{-}H]^-$ = 197 *m/z* in PA100, PA95, PA85 and in STD solution for comparison, from bottom to top, respectively, [a] identified with STD, [b] tentatively assigned.

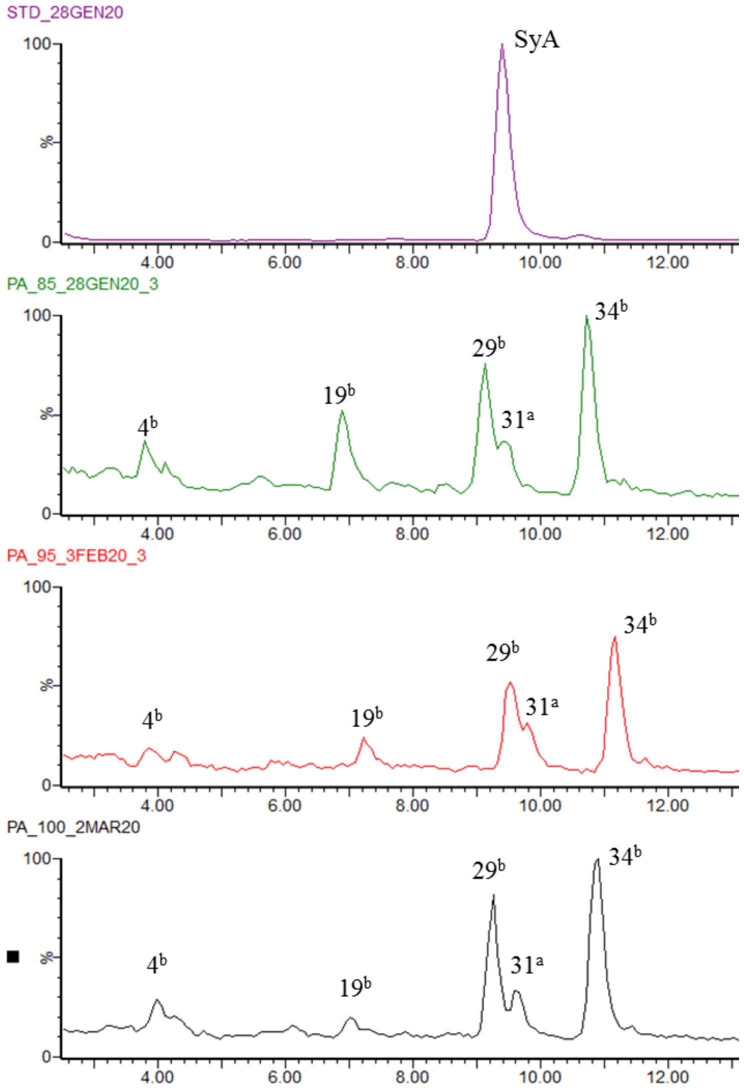

Figure S6. ES- channel of the anion $[M-H]^-$ = 353 *m/z* in PA100, PA95, PA85 and in STD solution for comparison, from bottom to top, respectively, [a] identified with STD, [b] tentatively assigned.

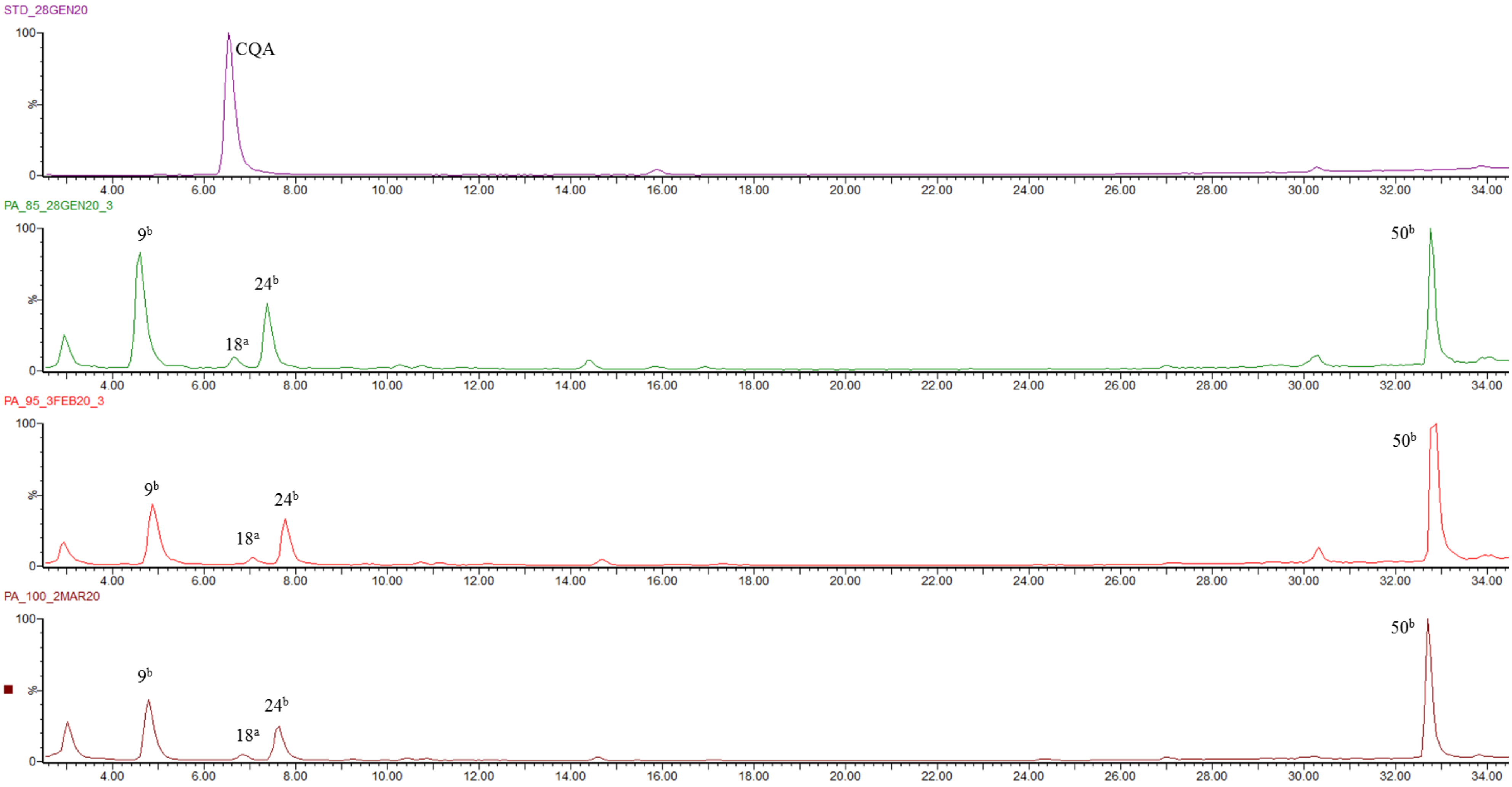

Figure S7. ES- channel of the anion $[M-H]^-$ = 179 *m/z* in PA100, PA95, PA85 and in STD solution for comparison, from bottom to top, respectively, [a] identified with STD, [b] tentatively assigned.

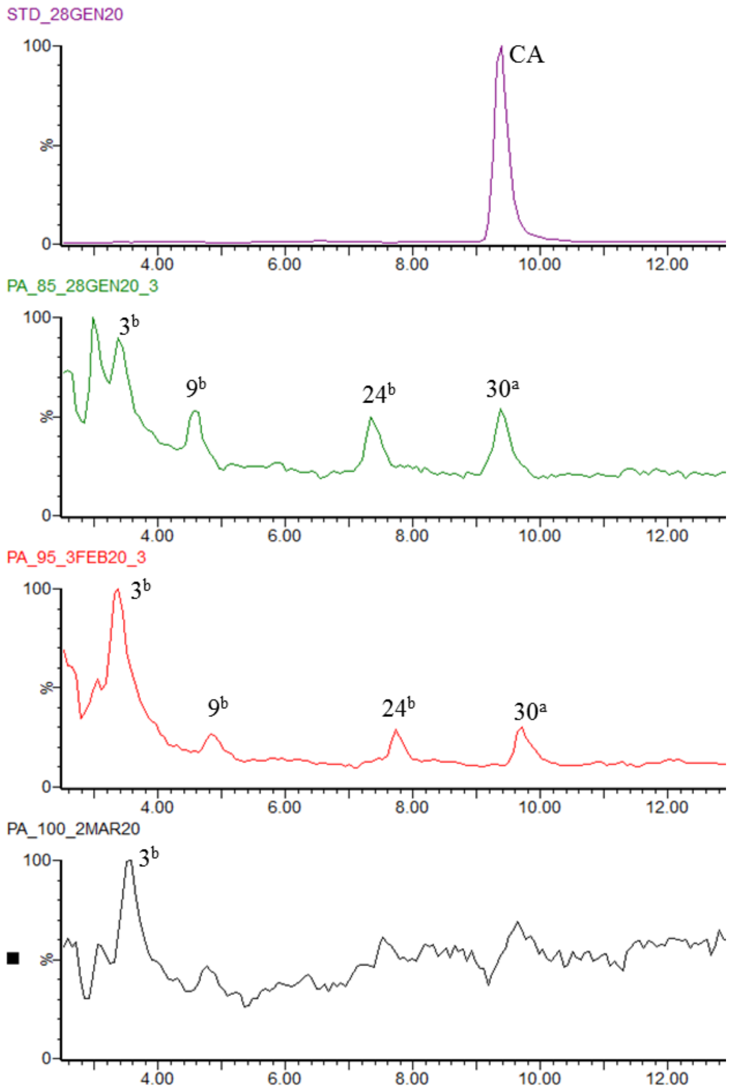

Figure S8. ES- channel of the anion $[M\text{-}H]^-$ = 163 *m/z* in PA100, PA95, PA85 and in STD solution for comparison, from bottom to top, respectively, [a] identified with STD, [b] tentatively assigned.

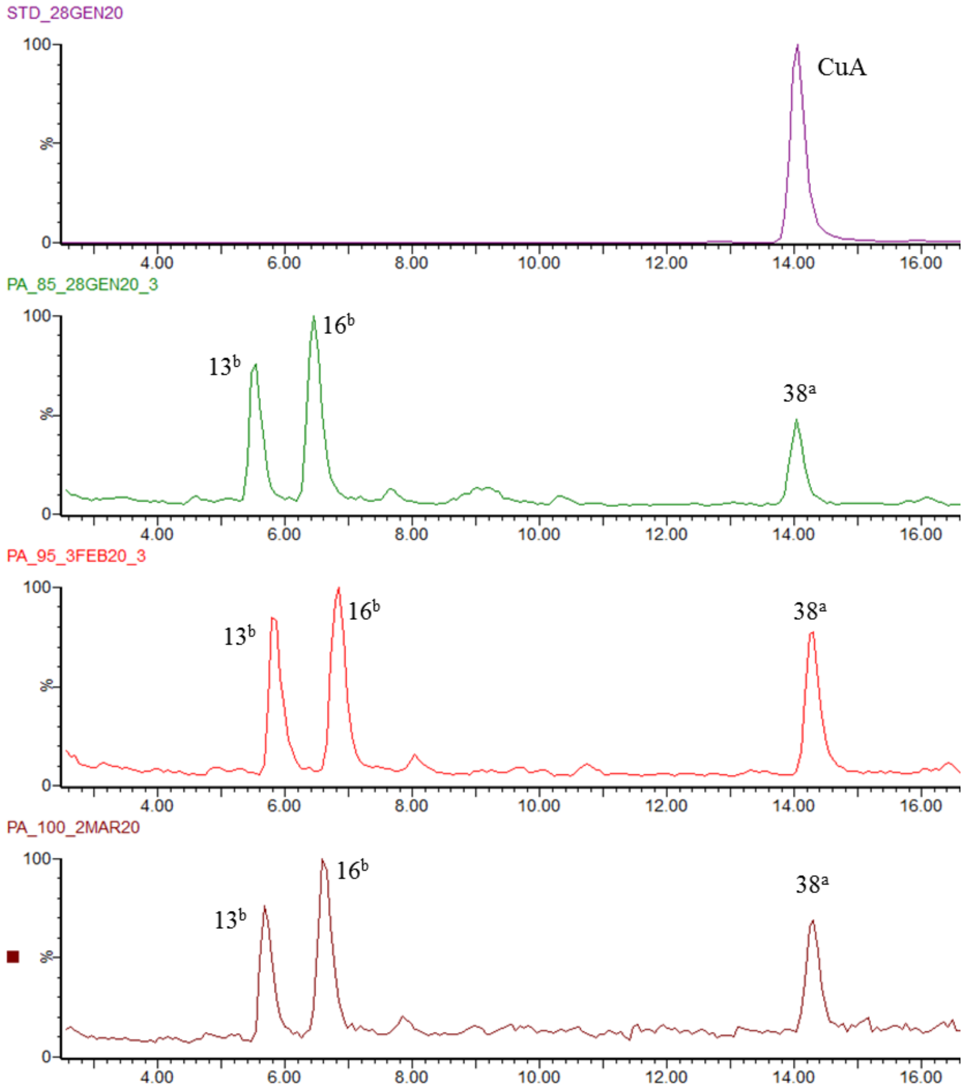

Figure S9. ES- channel of the anion $[M-H]^-$ = 193 *m/z* in PA100, PA95, PA85 and in STD solution for comparison, from bottom to top, respectively, [a] identified with STD, [b] tentatively assigned.

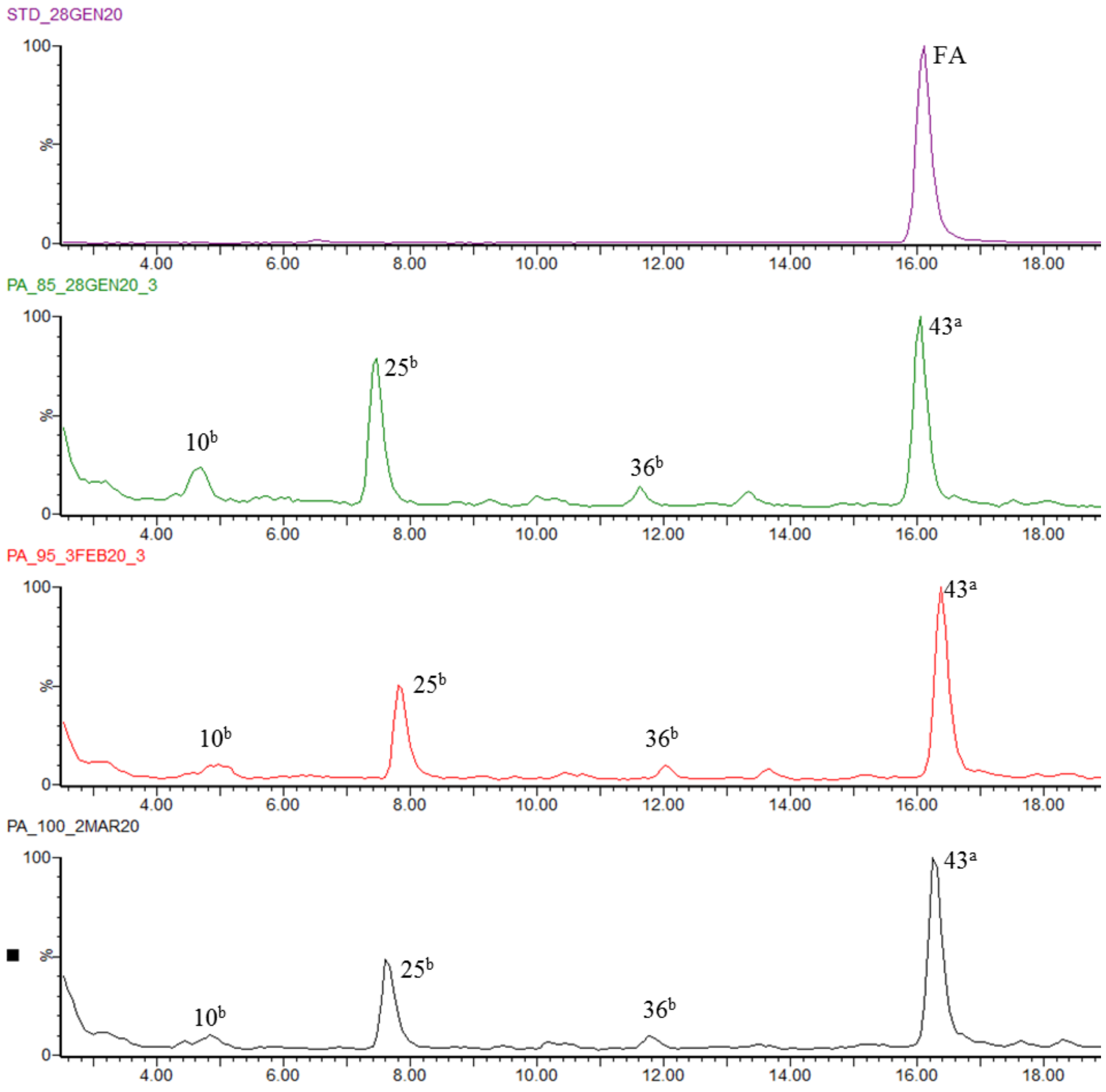

Figure S10. ES- channel of the anion $[M-H]^-$ = 223 *m/z* in PA100, PA95, PA85 and in STD solution for comparison, from bottom to top, respectively, [a] identified with STD, [b] tentatively assigned.

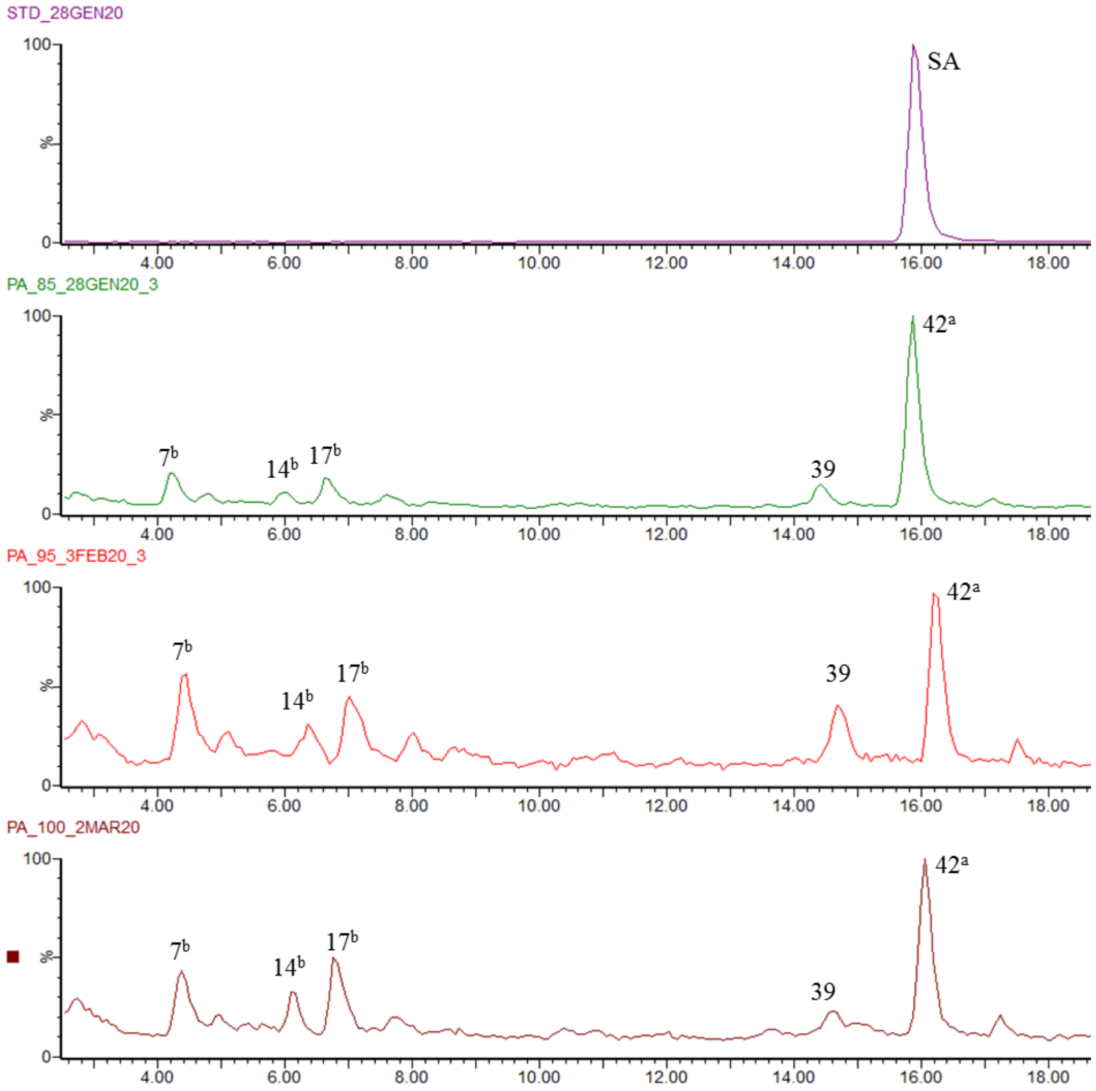

Figure S11. ES- channel of the anion [M-H]$^-$ = 609 *m/z* in PA100, PA95, PA85 and in STD solution for comparison, from bottom to top, respectively, [a] identified with STD, [b] tentatively assigned.

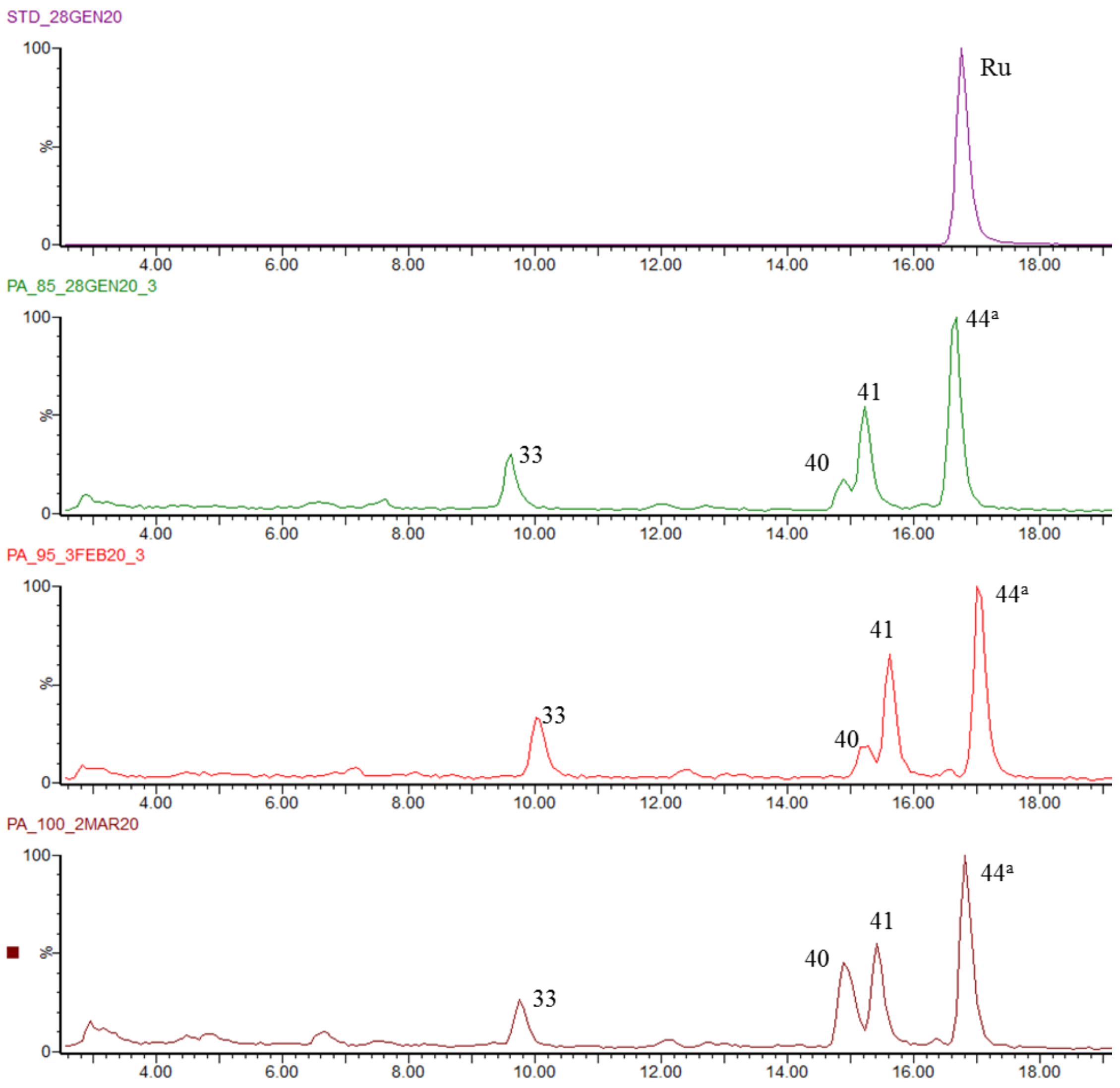

Figure S12. ES- channel of the anion $[M-H]^-$ = 301 *m/z* in PA100, PA95, PA85 and in STD solution for comparison, from bottom to top, respectively, [a] identified with STD, [b] tentatively assigned.

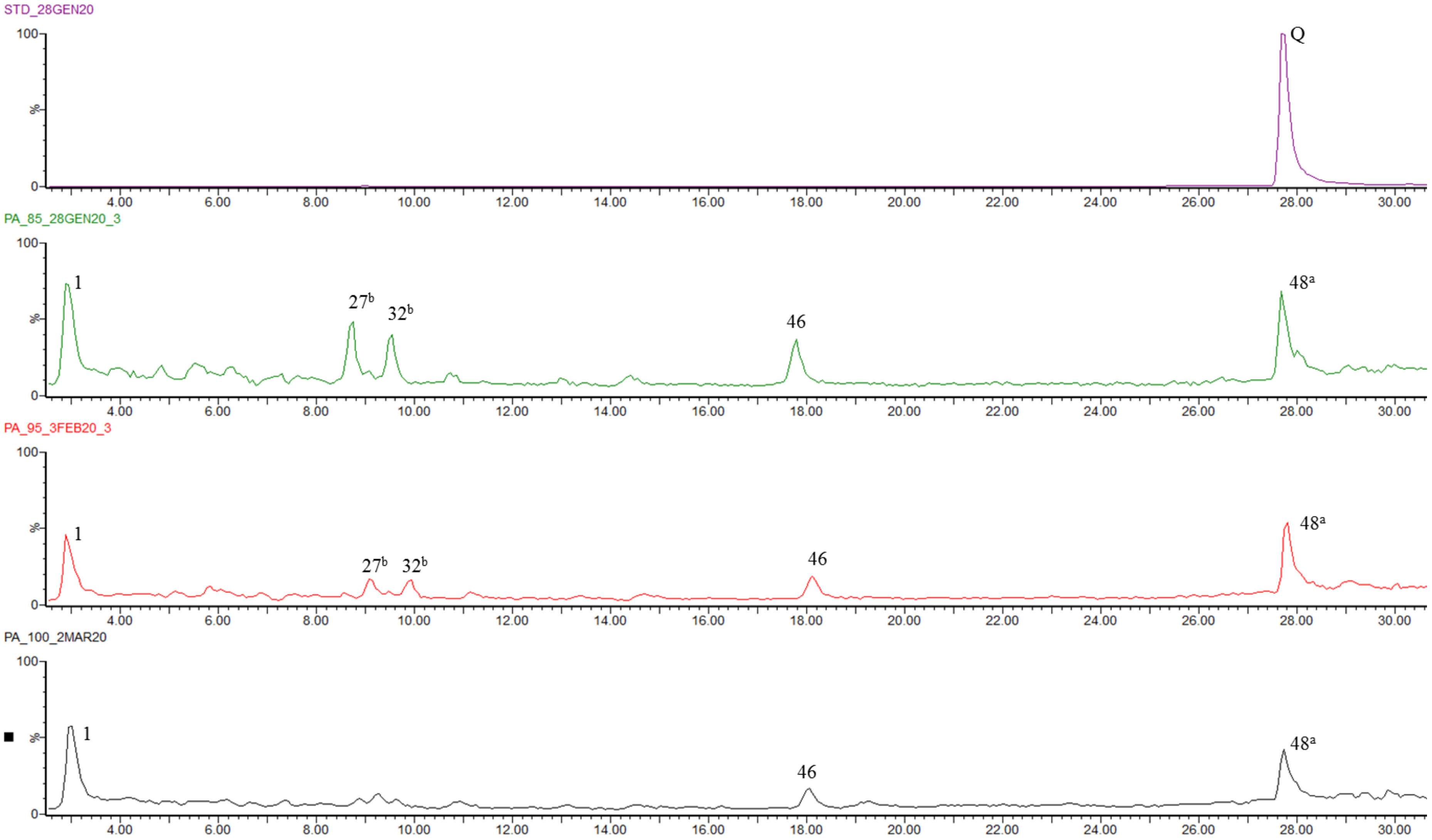

Figure S13. ES- channel of the anion $[M-H]^-$ = 285 *m/z* in PA100, PA95, PA85 and in STD solution for comparison, from bottom to top, respectively, [a] identified with STD, [b] tentatively assigned.

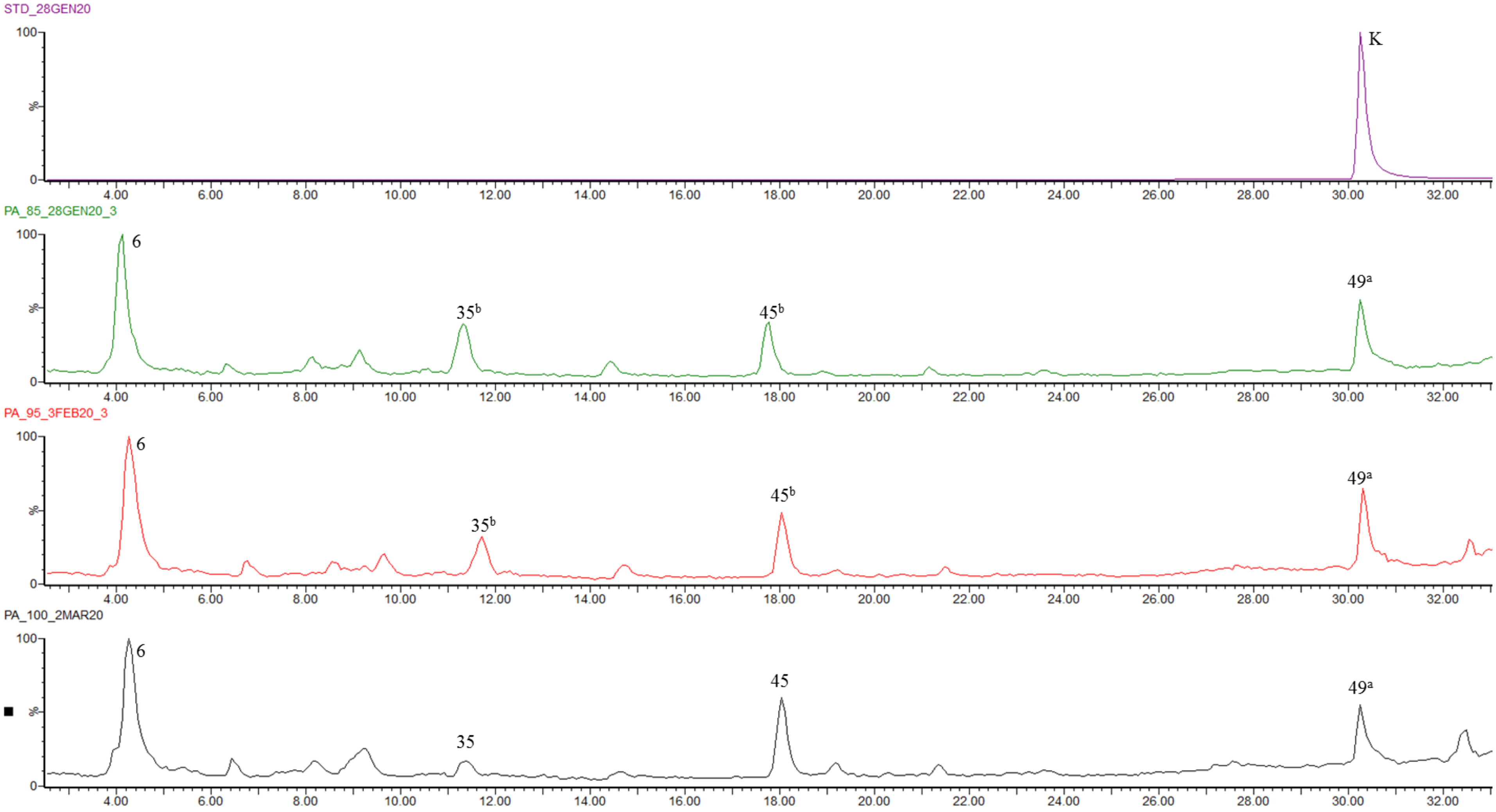

Figure S14. ES- channel for GA (*m/z* 169), PCA (*m/z* 153), pHBA (*m/z* 137), VA (*m/z* 167), CA (*m/z* 179), and SyA (*m/z* 197), from bottom to top, in 1:10 diluted LA-PA85.

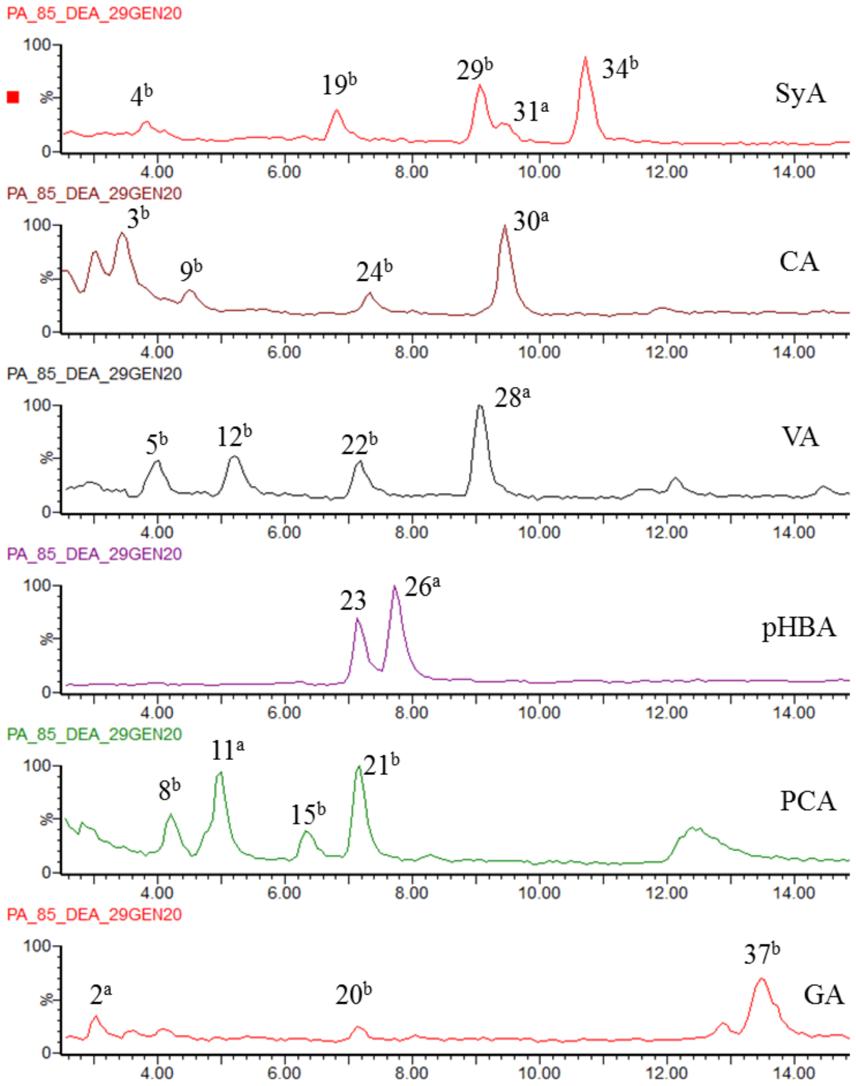

Figure S15. ES- channel for CuA (*m/z* 163), SA (*m/z* 223), FA (*m/z* 193), and Ru (*m/z* 609), in 1:10 diluted LA-PA85.

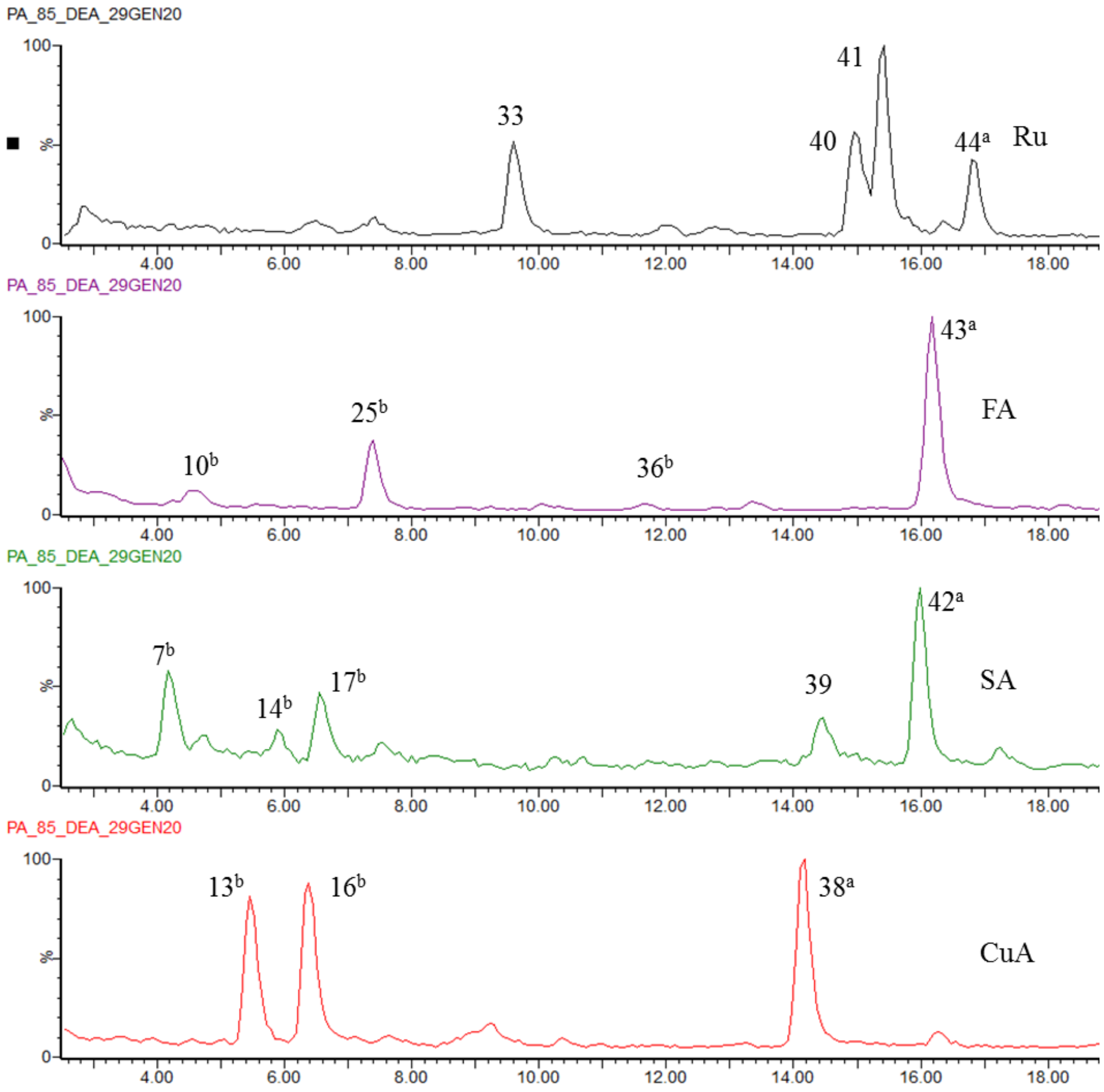

Figure S16. ES- channel for CQA (*m/z* 353), Q (*m/z* 301), and K (*m/z* 285), in 1:10 diluted LA-PA85.

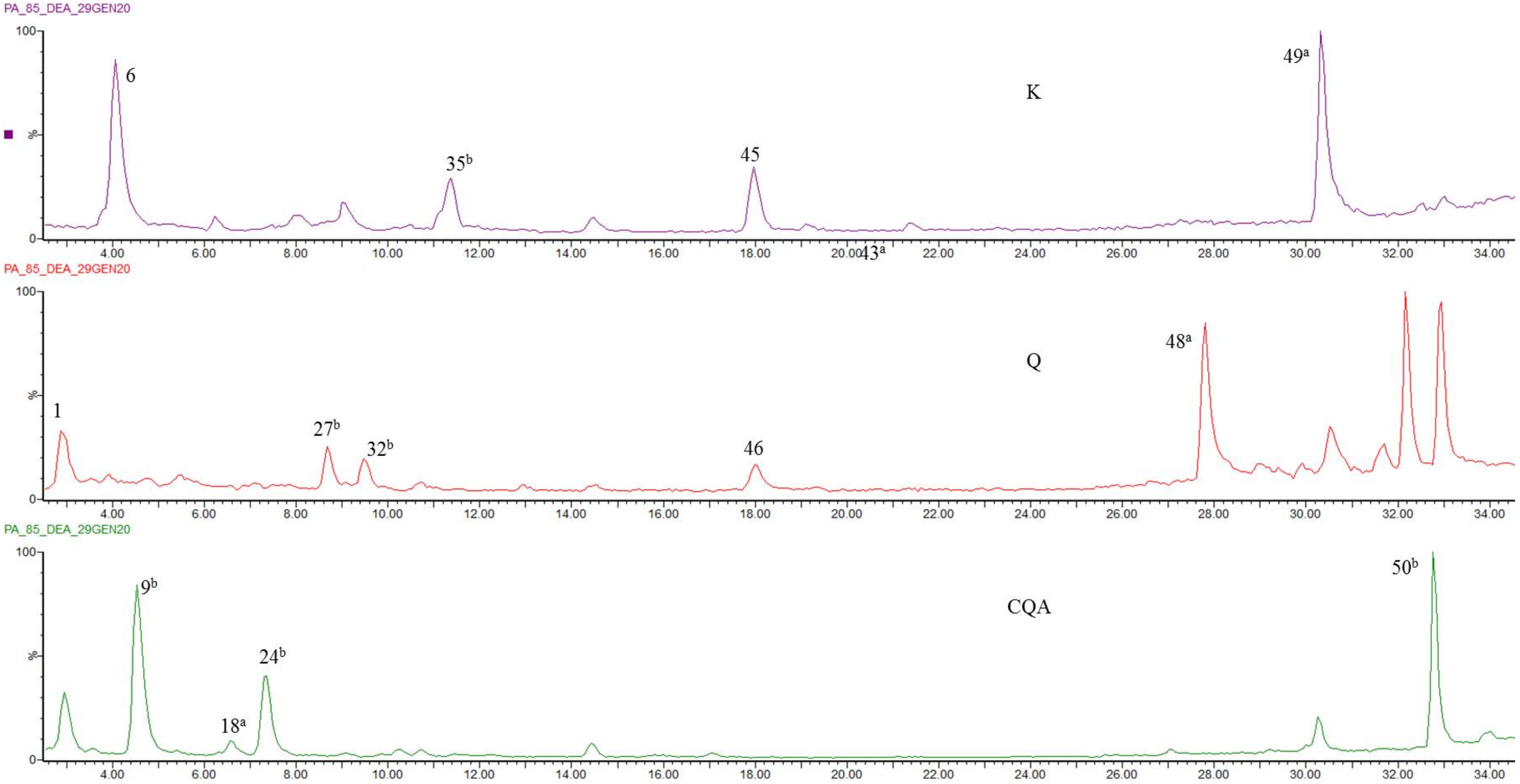

Figure S17. 600 MHz ^{1}H NMR spectrum of PA100; and assignments: **1**, lactic acid; **2**, iso-pentanol; **3**, alanine; **4**, propanol; **5**, iso-butanol; **6**, proline; **7**, acetic acid; **8**, pyruvic acid; **9**, pyroglutamic acid; **10**, succinic acid; **11**, 2-phenylethanol; **12**, choline; **13**, glycerophosphocholine; **14**, glycerol; **15**, maltodextrines α-glucose (1-6) units; **16**, maltodextrines α-glucose (reduced end) units; **17**, maltodextrines α-glucose (1-4) units; **18**, fumaric acid; **19**, tyrosine; **20**, gallic acid; **21**, uridine; **22**, cytidine; **23**, histidine; **24**, adenosine.

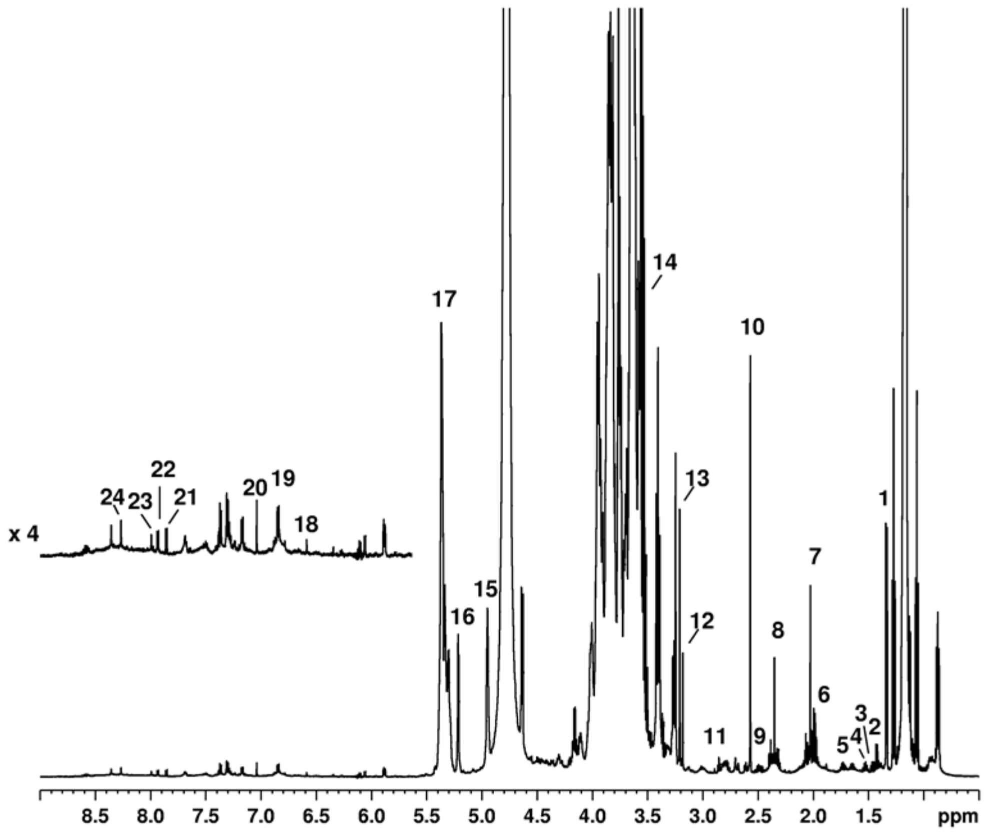

Supplement: Supplementary file 1 — jf1c00679_si_001.pdf [file jf1c00679_si_001.pdf]
